# Agreement testing of AMSTAR-PF, a tool for quality appraisal of systematic reviews of prognostic factor studies

**DOI:** 10.1136/bmjopen-2025-109388

**Published:** 2026-01-27

**Authors:** Michael L Henry, Neil E O’Connell, Richard D Riley, Karel G M Moons, Beverley J Shea, Lotty Hooft, Sarah B Wallwork, Johanna A A G Damen, Nicole Skoetz, Ruth P Appiah, Carolyn Berryman, Sophie M Crouch, Grace A Ferencz, Ashley R Grant, Katherine M Henry, Aleksandra M Herman, Emma L Karran, Indika Koralegedera, Hayley B Leake, Erin MacIntyre, Brendan Mouatt, Karma Phuentsho, Daniel A Van Der Laan, Ellana Welsby, Louise K Wiles, Erica M Wilkinson, Marelle K Wilson, Monique V Wilson, G Lorimer Moseley

**Affiliations:** 1 IIMPACT in Health, Adelaide University, Kaurna Country, Adelaide, South Australia, Australia; 2 Centre for Health and Wellbeing Across the Lifecourse, Department of Health Sciences, Brunel University London, London, UK; 3 Department of Applied Health Sciences, School of Health Sciences, College of Medicine and Health, University of Birmingham, Birmingham, UK; 4 National Institute for Health and Care Research (NIHR) Birmingham Biomedical Research Centre, Birmingham, UK; 5 Cochrane Netherlands, University Medical Center Utrecht, Utrecht University, Utrecht, Netherlands; 6 Julius Center for Health Sciences and Primary Care, University Medical Center Utrecht, Utrecht University, Utrecht, Netherlands; 7 School of Epidemiology and Public Health, Faculty of Medicine, University of Ottawa, Ottawa, Ontario, Canada; 8 Bruyere Health Research Institute, University of Ottawa, Ottawa, Ontario, Canada; 9 Institute of Public Health, University of Cologne, Cologne, Germany; 10 Brain Stimulation, Imaging and Cognition Group, Adelaide University, Adelaide, South Australia, Australia; 11 Princess Alexandra Hospital, Woolloongabba, Queensland, Australia; 12 Nencki Institute of Experimental Biology Polish Academy of Sciences, Warsaw, Poland; 13 Persistent Pain Research Group, Hopwood Centre for Neurobiology, South Australian Health and Medical Research Institute (SAHMRI), Adelaide, South Australia, Australia

**Keywords:** STATISTICS & RESEARCH METHODS, Prognosis, Systematic Review

## Abstract

**Abstract:**

**Objectives:**

To test the agreement and usability of a novel quality appraisal tool: A MeaSurement Tool to Assess systematic Reviews of Prognostic Factor studies (AMSTAR-PF).

**Design:**

Observational study.

**Participants:**

14 appraisers of varied experience levels and backgrounds, including undergraduate, master’s and PhD students, postgraduate researchers, research fellows and clinicians.

**Study procedure:**

Eight systematic reviews were rated by all reviewers using AMSTAR-PF.

**Outcome measures:**

Planned measures included intrapair and inter-pair agreement using Cohen’s and Fleiss’ kappa, time of use and time to reach consensus. Interrater agreement was an added measure, and Gwet’s agreement coefficient was calculated and presented due to its greater stability across agreement levels. The percentage of intrapair agreements identical or one category apart was also presented.

**Results:**

Interrater agreement averaged 0.59 (range 0.21–0.90), inter-pair agreement 0.61 (range 0.24–0.91) and intrapair agreement 0.75 (range 0.45–0.95) across the domains, with agreement for the overall rating 0.46 (95% CI 0.30 to 0.62) for interrater agreement, 0.46 (95% CI 0.17 to 0.74) for inter-pair agreement and 0.68 (range of averages 0.22–1.00) for intrapair agreement. The majority (60.7%) of intrapair ratings were identical, with 94.6% of final ratings either identical or only one category different for the overall appraisal. The time taken to appraise a study with AMSTAR-PF improved with use and averaged around 34 min after the first two appraisals.

**Conclusions:**

Despite some variance in agreement for different domains and between different appraisers, the testing results suggest that AMSTAR-PF has clear utility for appraising the quality of systematic reviews of prognostic factor studies.

**STRENGTHS AND LIMITATIONS OF THIS STUDY**

- The testing protocol was preregistered and standardised across all appraisers.
- The 14 appraisers, who had varying levels of experience, tested A MeaSurement Tool to Assess systematic Reviews of Prognostic Factor studies on eight articles covering a range of topics.
- Gwet’s agreement coefficient and kappa values were calculated across interrater, inter-pair and intrapair agreement, and time of use and time to consensus were recorded.
- Appraisers had limited experience in prognostic factor research and reviews were often outside their expertise.

## Introduction

 There is an increasing number of studies and systematic reviews investigating prognostic factors (PFs).^1 2^ PFs are variables associated with (the risk or value) of future outcomes^3^ and are particularly useful in providing patients with a prognosis and developing prognostic models (eg, for outcome risk prediction). Nomenclature for PFs is not consistent in the medical literature, and sometimes PFs are also known as predictors, risk factors or prognostic variables/determinants/covariates. Importantly, our focus is not on whether factors are predictive of treatment effect (sometimes called predictive markers, especially in the cancer field); rather, we are solely focused on factors that are associated with future outcomes (irrespective of how it impacts any treatment effect).

PFs can include patient demographics, such as age and sex; clinical signs and symptoms, such as imaging results and severity of symptoms; and broader environmental factors such as location of residence. Published studies of prognosis research are variable in quality and subject to a range of potential biases that can impact study findings and decrease confidence in the results.^1 4^ As the body of primary prognosis research grows, so will the demand for high quality evidence syntheses. Until recently, there has been no standardised tool specifically developed to appraise the quality of reviews of PF studies. In order to fill this gap, our group developed A MeaSurement Tool to Assess systematic Reviews of Prognostic Factor studies (AMSTAR-PF),^5^ through a multistage process, by a team including people with expertise in PF reviews, evidence synthesis and quality appraisal tool development.

AMSTAR-PF was based on AMSTAR 2,^6^ a quality appraisal tool designed for systematic reviews of interventional research. AMSTAR-PF consists of 14 domains, some of which have subsections, resulting in 19 specific questions that inform an overall judgement on the quality of the review (see online supplemental appendix 1 in Supplementary material). All 19 questions have answer options of Yes (Y), Probably Yes (PY), Probably No (PN) and No (N). Six questions also have an option of Not Applicable (N/A). The final judgement has four options for the overall quality of the review: High, Moderate, Low and Critically Low. As part of the development process, we sought to test AMSTAR-PF to determine measures of agreement and usability.

Agreement relates to the reliability of a tool and has two main subdivisions; how consistently a given item is rated by the same rater (or same group of raters) on different occasions, and how similarly two or more different raters (or groups) rate the same item, which is the agreement we were interested in for this paper. Usability of a tool can consider reported difficulties or ease of use, as well as objective measures such as time to use. In this paper, we aimed to determine agreement and usability of AMSTAR-PF, using (1) measures of interrater, inter-pair and intrapair agreement, and (2) time to complete appraisal using the tool by researchers with a diverse range of research experience.

## Methods

### Registration

We preregistered our protocol on OSF prior to testing (osf.io/acrwf).

### Patient and public involvement

Patients and members of the public were not involved in this methodological research.

### Testing procedure

**Figure 1:**
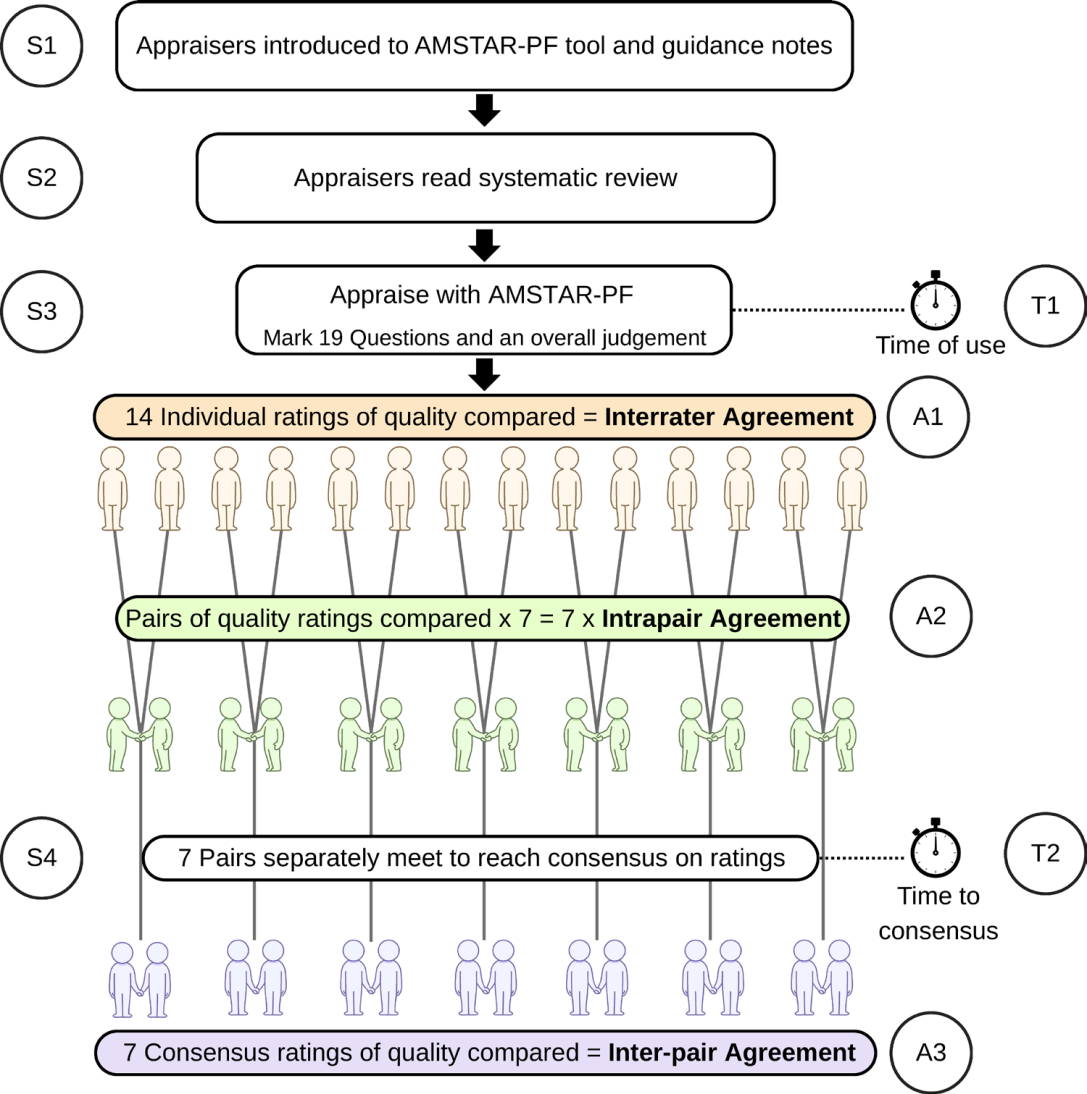
Schematic of testing procedure, showing the steps involved (S1–4) for appraisers, the types of agreement (A1–3) and the timings assessed (T1–2). First, all appraisers were introduced to AMSTAR-PF (S1). Each appraiser then read the systematic review (S2) and timed themselves while they used AMSTAR-PF to appraise the article, completing all 19 questions and the overall judgement of review quality (S3). This gave a time of use (T1). The ratings for each question and overall quality were compared across the 14 appraisers to give interrater agreement (A1). These ratings were also compared within each of the seven preallocated pairs to give seven scores of intrapair agreement (A2). The pairs met up (after the first two articles, and then at the end), and timed themselves reaching consensus on each of the 19 questions and overall appraisal (S4), giving a time to consensus (T2). The seven pairs’ scores for the 19 questions and overall rating were compared to give inter-pair agreement (A3). AMSTAR-PF, A MeaSurement Tool to Assess systematic Reviews of Prognostic Factor studies.

The testing procedure is outlined in figure 1. Testing used a convenience sample of eight systematic reviews of PF studies. Each systematic review will here be referred to as an ‘article’. The selected articles included two in low back pain,^7 8^ two in cancer,^9 10^ two in brain injury^11 12^ and two in COVID-19,^13 14^ and included meta-analyses and narrative syntheses. Two articles^7 10^ were Cochrane reviews. The articles were selected to cover a range of different clinical areas, though the choice of the four areas was somewhat arbitrary. Back pain and concussion were topics of an umbrella review being undertaken by some of the research group. Cancer research was deemed likely to have a selection of PF reviews, and COVID-19, being a newly emerged disease, was chosen to represent current practice in PF research. The two Cochrane reviews were selected from the Cochrane Library website, with the prognosis filter applied, to match one of the four topics. The remaining articles were selected based on their titles from a Google Scholar search, and ensuring a mix of reviews with and without meta-analyses.

Testing of the tool was completed in March 2024 by appraisers who were independent of the process of developing AMSTAR-PF and who had not been involved in any of the preliminary pilot testing stages. Appraisers were recruited via an email sent to 43 people associated with a university research group. We limited the number of appraisers to 14 for pragmatic reasons; this was the number of people who were interested and available during the proposed testing period. Each of the eight articles was appraised by the 14 appraisers (seven pairs). Where possible, pairs consisted of researchers with different experience levels (eg, a student with a postdoctoral researcher), because we considered that this would most faithfully reflect the likely use of the tool in research practice.

### Agreement

All appraisers were provided with the AMSTAR-PF tool and Guidance Notes^5^ and asked to familiarise themselves with these documents prior to assessing the articles. Appraisers had an opportunity to ask questions and seek further clarification from the lead author (MLH), if needed. Each appraiser then independently applied the AMSTAR-PF tool on two articles—one Cochrane review article and one non-Cochrane article. Appraiser pairs met to discuss their appraisal, reach consensus on these first two articles, discuss consistency in the application of the tool and develop any decision-making rules they would implement, as a pair, for the remainder of the articles. This practice is recommended^15^ and often used when undertaking quality appraisals of articles and reflects how the tool is intended to be used. Once the pair was satisfied with their consensus, each appraiser independently appraised the remaining six articles. When both appraisers in a pair had completed the appraisals for the remaining six articles, they again compared findings and reached consensus for these six articles. For all eight articles, the overall rating for each article as well as the responses to each question within AMSTAR-PF were compared; agreements and discrepancies were recorded.

### Timing

For each of the articles, appraisers were asked to read the article first and then record the time from the start of the AMSTAR-PF appraisal process to completion. Each appraiser pair also recorded the total time taken to reach consensus for each of the articles, which consisted of consensus on the overall rating as well as each of AMSTAR-PF’s 19 questions. Appraisers were asked to record and describe any instances where they could not reach consensus.

To mitigate any effect of order on ratings, agreement or timing, we counterbalanced the order of the eight articles across appraiser pairs, with three pairs randomised to complete one sequence (Aldin *et al*,^10^ Izcovich *et al*,^13^ Maglietta *et al*,^14^ Mercier *et al*,^12^ Pinheiro *et al*,^8^ Puig *et al*,^11^ Wijnands *et al*,^9^ Hayden *et al*^7^) and four pairs completing the reverse sequence.

### Analysis

Interrater agreement (the agreement across all 14 appraisers), inter-pair agreement (the agreement across the consensus scores of the seven pairs) and intrapair agreement (the mean of the agreement between the two members of each of the seven pairs) were calculated across the eight articles, both overall and for each of the individual questions. The decision to add interrater agreement, for extra detail, was made after the protocol was registered. We chose the analyses based on measures that we believed would be most important for potential users of the tool. A common reason for appraising the quality of a systematic review is as part of an umbrella review. In most umbrella reviews, quality appraisal will be performed independently by two reviewers, with the consensus score being the final, accepted rating of quality. As such, the intrapair agreement is likely to be relevant for review teams in most situations, and the inter-pair agreement is most relevant for comparing different review teams who have assessed the same paper. The interrater agreement was added as another metric but is perhaps less practically relevant—it is rarer that a single person’s appraisal will be published, and so comparing many different individuals’ ratings would then be less likely. For the domain questions, we calculated agreement with the original answering options (Y, PY, PN and N, and for some questions, N/A), as well as with the Y/PY and N/PN answers collapsed, as per our protocol.

We used Gwet’s agreement coefficient (AC) in our analysis of the agreement. This was a deviation from our original protocol, which stipulated using Cohen’s (for two raters) and Fleiss’ kappa (for more than two raters) for analyses. We made this deviation after data collection but prior to commencing any analysis, after receiving statistical advice. Gwet’s AC is a chance-corrected AC that was developed in part due to instability of kappa statistics across a range of agreement levels, in particular paradoxes in which a high level of agreement can lead to low kappa scores, and is becoming more recommended in a range of settings.^16^,^25^ Gwet’s AC can be used with two or more raters and has been shown to provide stable measures of agreement across a range of agreement levels. Furthermore, although kappa and Gwet’s AC are used in agreement testing, it has been argued that Cohen’s kappa is an association test, rather than an agreement test.^26^ Gwet’s AC1 was calculated for nominal data (questions 2b, 7c, 9a, 9b, 10 and 12, which include an N/A option), while Gwet’s AC2, with a linear weighting, was used for the ordinal data (the remaining 13 questions and the overall judgement).^27^ Consistent with our original protocol, we have also calculated Cohen’s and Fleiss’ kappa, which can be found in online supplemental appendix 4 of the supplementary material. Calculations were performed within Stata/SE V.18.0 (StataCorp LLC).

Interpretations of the ACs were based on Landis and Koch’s benchmarks^28^ and calculated in the manner recommended by Gwet.^23 27^ This manner considers the SE to give a probability of the value falling within a certain benchmark category, rather than simple alignment of the point estimate. We used the recommended 95% cumulative probability as the cut-off level.^27^ To calculate this, the probability of the agreement falling within each benchmark band is calculated, and the probabilities summed starting with the top band (0.8–1.0) and working down. Once the cumulative probability reaches 95% or higher, then that band is the one used as the benchmark interval.^23 27^ RStudio V.2024.04.1 Build 748 (RStudio) and the irrCAC package were used for calculations, and ggplot or Microsoft Excel (V.2408, Microsoft Corporation) for visualisation. The category labels for Gwet’s AC were aligned with Landis and Koch’s suggestions: <0, poor; 0.0–0.2, slight; 0.2–0.4, fair; 0.4–0.6, moderate; 0.6–0.8, substantial; and 0.8–1.0, almost perfect.^28^

We also presented the raw numbers of agreements and disagreements for each of the intrapair decisions. This was a deviation from protocol, but we believe it adds interpretability and clarity.

Descriptive statistics were calculated for ‘time taken to use the tool’, and ‘time taken to reach consensus’.

To detect an effect of the consensus process or learning on level of agreement, we compared agreement for the first two articles appraised to agreement for the next six, and agreement for the first two articles to agreement for the final two. We also investigated whether there were differences in agreement between the two different orders of articles. We did this by comparing Gwet’s AC for the two orders, and by comparing the final ratings of each article. Furthermore, we compared agreement for the Cochrane articles to those for the non-Cochrane articles. The timing of completion was compared for the order of completion, as well as for the experienced (seven researchers with five or more years in research) versus novice (seven researchers with three or fewer years in research) researchers within the 14 appraisers.

To detect group differences in agreement and timings, we used paired t-tests when data were normally distributed, and Wilcoxon signed-rank or rank-sum tests when they were not. A significance level of p<0.05 was used.

## Results

### Demographics of appraisers

The 14 appraisers (11 female, 3 male) were aged between 22–59 years old and had experience in research ranging from less than 1 year to over 10 years. Appraisers included Honours, Masters and PhD students, postdoctoral researchers and research fellows. Some appraisers also worked clinically. 11 spoke English as their first language and three as an additional language. One appraiser had previous experience with PF research. 12 had experience with pain research. None had research experience with any of the other three topics addressed in the articles (COVID-19, brain injury and cancer). 12 had experience of performing risk of bias and/or quality appraisal assessments, including 3 who had used AMSTAR/AMSTAR 2.

### Interrater, inter-pair and intrapair agreement across the domains of the AMSTAR-PF

**Figure 2:**
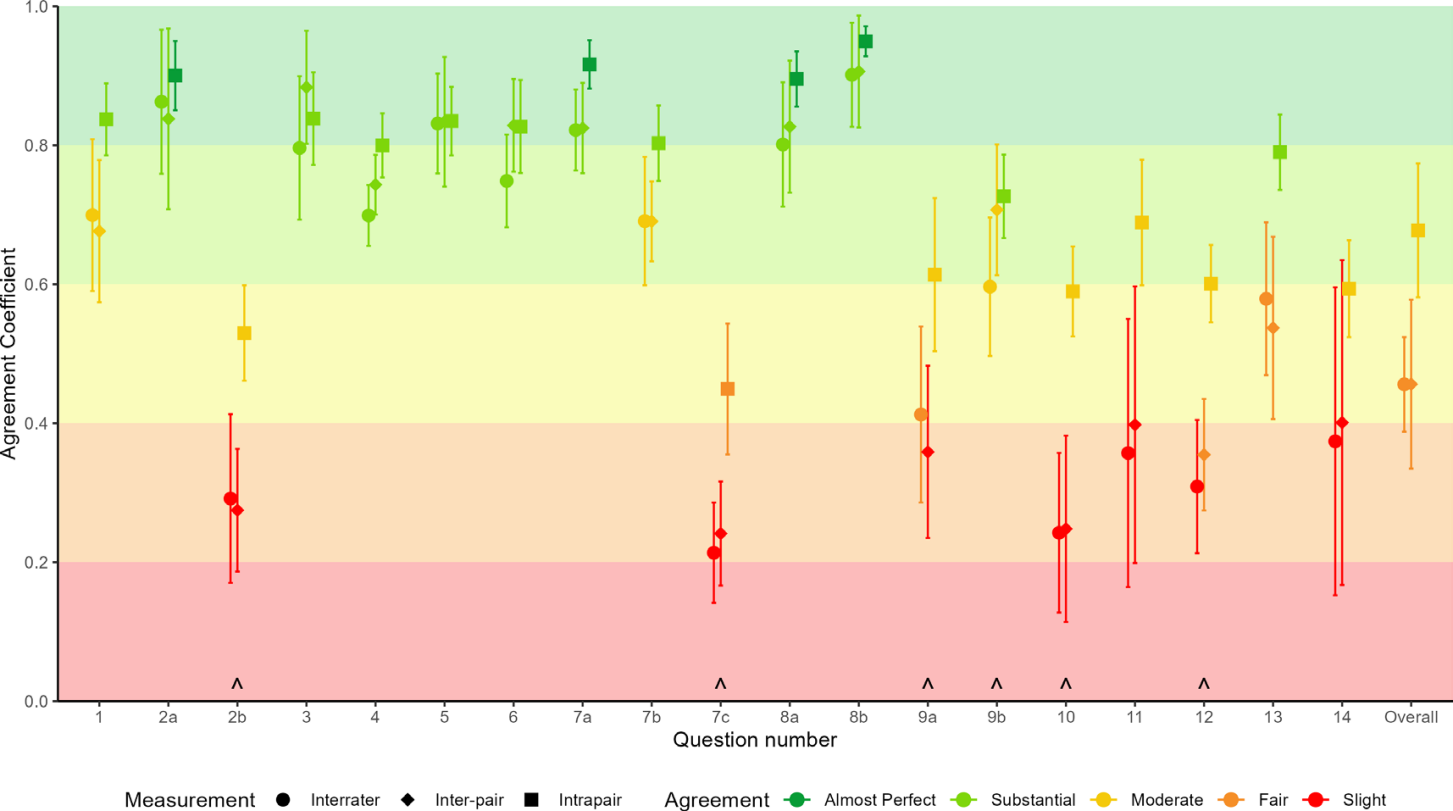
Gwet’s AC for each AMSTAR-PF question, with all answering options and for the overall appraisal. Interrater and inter-pair show AC and SE as error bars. Intrapair show average AC across pairs with SEM as the error bars. Questions marked (∧) had an N/A option and were treated as nominal data, calculated as Gwet’s AC1. All other questions were calculated as Gwet’s AC2 with linear weighting. Benchmark interpretation is colour-coded, with each plotted point coloured according to the calculated 95% cumulative probability for Landis and Koch’s benchmark categories; <0, poor; 0.0–0.2, slight; 0.2–0.4, fair; 0.4–0.6, moderate; 0.6–0.8, substantial; and 0.8–1.0, almost perfect. AC, agreement coefficient; AMSTAR-PF, A MeaSurement Tool to Assess systematic Reviews of Prognostic Factor studies; N/A, not applicable.

Interrater agreement across the 14 appraisers ranged between slight and substantial (Gwet’s AC mean 0.59, 95% CI 0.48 to 0.70, range 0.21–0.90) for the 19 questions of AMSTAR-PF, and inter-pair agreement also ranged between slight and substantial (mean 0.61, 95% CI 0.49 to 0.73, range 0.24–0.91). Intrapair agreement was taken as the mean of the seven intrapair AC scores and ranged between fair to almost perfect agreement (mean 0.75, 95% CI 0.68 to 0.82, range of means 0.45–0.95). See table 1 and figure 2 for more detailed information. Of the 1064 domain intrapair decisions, 776 (72.9%) were identical, and 197 (18.5%) were one category different, meaning that 8.6% of intrapair decisions differed by more than one category, or involved one appraiser in a pair selecting the N/A option and the other member of the pair deeming the question applicable.

**Table 1:** Gwet’s AC for each AMSTAR-PF question, with all answering options and for the final judgement

Question	Interrater		Inter-pair		Intrapair	
	AC (95% CI)	I	AC (95% CI)	I	AAC (95% CI)	I
**1** Research question	0.70 (0.44 to 0.96)		0.68 (0.43 to 0.92)		0.84 (0.71 to 0.96)	
**2a** Protocol registration	0.86 (0.62 to 1.00)		0.84 (0.53 to 1.00)		0.90 (0.78 to 1.00)	
**∧2b** Deviations from protocol	0.29 (0.00 to 0.58)		0.27 (0.07 to 0.48)		0.53 (0.36 to 0.70)	
**3** Included study designs	0.80 (0.55 to 1.00)		0.88 (0.69 to 1.00)		0.84 (0.68 to 1.00)	
**4** Search strategy	0.70 (0.60 to 0.80)		0.74 (0.64 to 0.84)		0.80 (0.67 to 0.91)	
**5** Inclusion process	0.83 (0.66 to 1.00)		0.83 (0.61 to 1.00)		0.83 (0.71 to 0.96)	
**6** Excluded studies	0.75 (0.59 to 0.91)		0.83 (0.67 to 0.99)		0.83 (0.66 to 0.99)	
**7a** Data extraction	0.82 (0.68 to 0.96)		0.82 (0.67 to 0.98)		0.92 (0.83 to 1.00)	
**7b** Description of studies	0.69 (0.47 to 0.91)		0.69 (0.55 to 0.83)		0.80 (0.67 to 0.94)	
**∧7c** PF effect calculations	0.21 (0.04 to 0.38)		0.24 (0.06 to 0.42)		0.45 (0.22 to 0.68)	
**8a** RoB process	0.80 (0.59 to 1.00)		0.83 (0.60 to 1.00)		0.90 (0.80 to 0.99)	
**8b** RoB technique	0.90 (0.72 to 1.00)		0.91 (0.72 to 1.00)		0.95 (0.90 to 1.00)	
**∧9a** Synthesis interpretability	0.41 (0.11 to 0.71)		0.36 (0.07 to 0.65)		0.61 (0.34 to 0.88)	
**∧9b** Meta-analysis	0.60 (0.36 to 0.83)		0.71 (0.48 to 0.93)		0.73 (0.58 to 0.87)	
**∧10** Small study effects	0.24 (−0.03 to 0.51)		0.25 (−0.07 to 0.56)		0.59 (0.43 to 0.75)	
**11** Impact of RoB	0.36 (−0.10 to 0.81)		0.40 (−0.07 to 0.87)		0.69 (0.47 to 0.91)	
**∧12** Heterogeneity	0.31 (0.08 to 0.54)		0.35 (0.17 to 0.54)		0.60 (0.46 to 0.74)	
**13** Conflicts of interest	0.58 (0.32 to 0.84)		0.54 (0.23 to 0.85)		0.79 (0.66 to 0.92)	
**14** Certainty of findings	0.37 (−0.15 to 0.90)		0.40 (−0.15 to 0.95)		0.59 (0.42 to 0.76)	
**Final appraisal**	**0.46 (0.30 to 0.62**)		**0.46 (0.17 to 0.74**)		**0.68 (0.44 to 0.91**)	

Questions marked (∧) had an N/A option and were treated as nominal data, calculated as Gwet’s AC1. All other questions were calculated as Gwet’s AC2 with linear weighting. CIs are capped at 1.00. Benchmark interpretation is calculated using 95% cumulative probabilities for Landis and Koch’s benchmark categories, 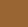
< 0, poor; 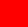 0.0–0.2, slight; 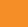
0.2–0.4, fair; 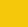-0.4–0.6, moderate; 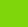
0.6–0.8, substantial; and 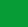
0.8–1.0, almost perfect.

AAC, Average Gwet's Agreement Coefficient; AC, Gwet’s agreement coefficient; AMSTAR-PF, A MeaSurement Tool to Assess systematic Reviews of Prognostic Factor studies; I, interpretation category; N/A, not applicable; PF, prognostic factor; RoB, risk of bias.

**Figure 3:**
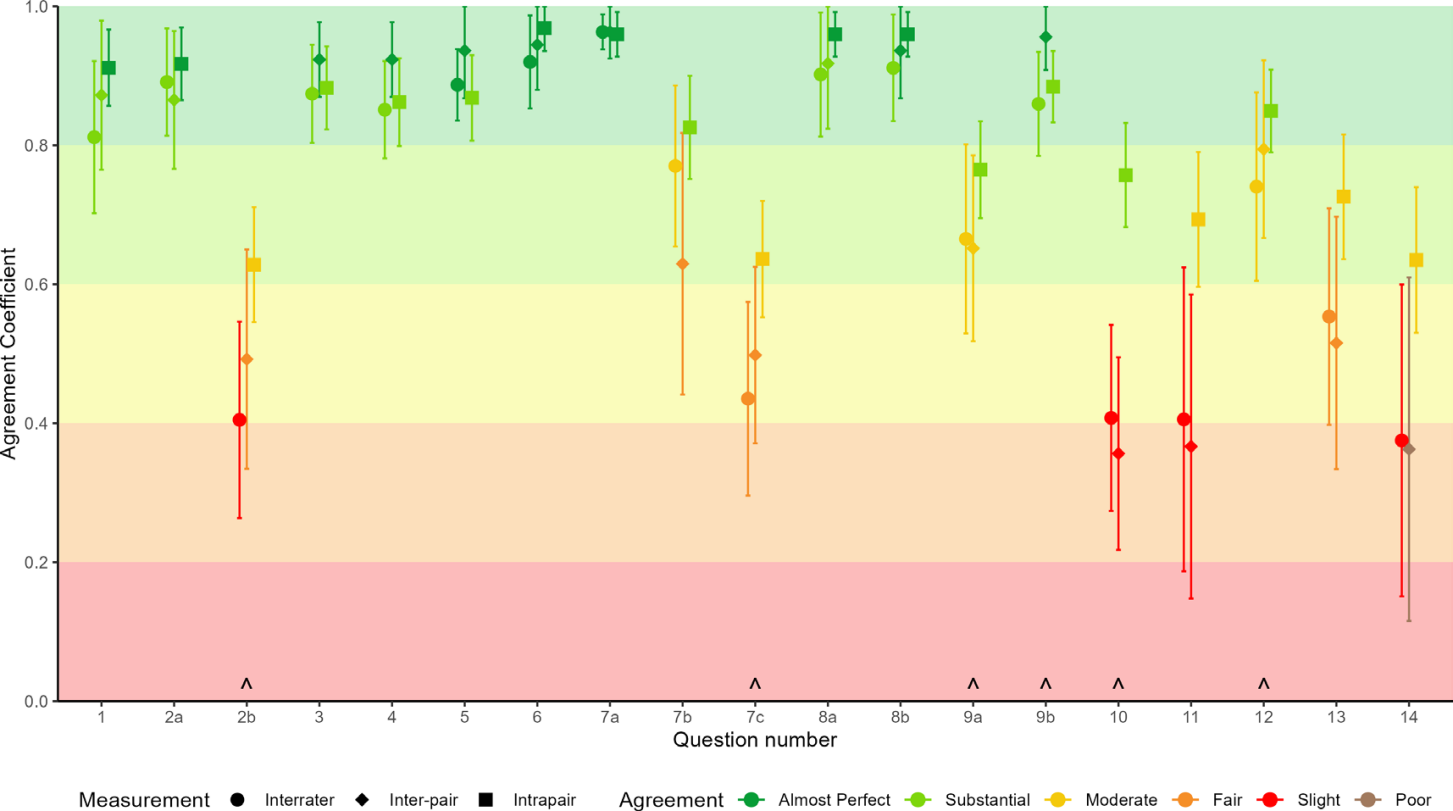
Gwet’s AC for each AMSTAR-PF question with dichotomised answering responses (Y/PY, and N/PN collapsed). Interrater and inter-pair show AC and SE as error bars. Intrapair show average AC across pairs with SEM as the error bars. Error Bars are capped at 1.0. Questions marked (∧) had an N/A option. Benchmark interpretation is colour-coded and calculated using 95% cumulative probabilities for Landis and Koch’s benchmark categories, <0, poor; 0.0–0.2, slight; 0.2–0.4, fair; 0.4–0.6, moderate; 0.6–0.8, substantial; and 0.8–1.0, almost perfect. AC, agreement coefficient; AMSTAR-PF, A MeaSurement Tool to Assess systematic Reviews of Prognostic Factor studies; N, no; N/A, not applicable; PN, partial no; PY, partial yes; Y, yes.

When answering options were collapsed (from ‘Yes and Partial Yes’, and ‘No and Partial No’ to ‘Yes’ and ‘No’, and in some questions, N/A), the interrater agreement over the 19 questions ranged from slight to almost perfect (0.38–0.96), the inter-pair agreement ranged between poor and almost perfect (0.36–0.96) and the mean intrapair agreements were between moderate and almost perfect (0.63–0.97). See table 2 and figure 3. Of the 1064 intrapair decisions, 934 (87.8%) were the same direction, or both members of the pair agreed it to be N/A.

**Table 2:** Gwet’s AC1 for dichotomised answering responses (Y/PY, and N/PN collapsed).

Question	Interrater		Inter-pair		Intrapair	
	AC (95% CI)	I	AC (95% CI)	I	AAC (95% CI)	I
**1** Research question	0.81 (0.55 to 1.00)		0.87 (0.62 to 1.00)		0.91 (0.76 to 1.00)	
**2a** Protocol registration	0.89 (0.71 to 1.00)		0.87 (0.63 to 1.00)		0.92 (0.77 to 1.00)	
**∧2b** Deviations from protocol	0.40 (0.07 to 0.74)		0.49 (0.12 to 0.87)		0.63 (0.37 to 0.88)	
**3** Included study designs	0.87 (0.71 to 1.00)		0.92 (0.80 to 1.00)		0.88 (0.70 to 1.00)	
**4** Search strategy	0.85 (0.69 to 1.00)		0.92 (0.80 to 1.00)		0.86 (0.74 to 0.99)	
**5** Inclusion process	0.89 (0.77 to 1.00)		0.94 (0.77 to 1.00)		0.87 (0.74 to 1.00)	
**6** Excluded studies	0.92 (0.76 to 1.00)		0.94 (0.79 to 1.00)		0.97 (0.89 to 1.00)	
**7a** Data extraction	0.96 (0.90 to 1.00)		0.96 (0.87 to 1.00)		0.96 (0.90 to 1.00)	
**7b** Description of studies	0.77 (0.50 to 1.00)		0.63 (0.18 to 1.00)		0.83 (0.64 to 1.00)	
**∧7c** PF effect calculations	0.44 (0.11 to 0.76)		0.50 (0.20 to 0.80)		0.64 (0.41 to 0.86)	
**8a** RoB process	0.90 (0.69 to 1.00)		0.92 (0.70 to 1.00)		0.96 (0.90 to 1.00)	
**8b** RoB technique	0.91 (0.73 to 1.00)		0.94 (0.77 to 1.00)		0.96 (0.90 to 1.00)	
**∧9a** Synthesis interpretability	0.67 (0.34 to 0.99)		0.65 (0.34 to 0.97)		0.76 (0.54 to 0.99)	
**∧9b** Meta-analysis	0.86 (0.68 to 1.00)		0.96 (0.84 to 1.00)		0.88 (0.74 to 1.00)	
**∧10** Small study effects	0.41 (0.09 to 0.72)		0.36 (0.03 to 0.68)		0.76 (0.56 to 0.96)	
**11** Impact of RoB	0.41 (−0.11 to 0.92)		0.37 (−0.15 to 0.88)		0.69 (0.43 to 0.95)	
**∧12** Heterogeneity	0.74 (0.42 to 1.00)		0.79 (0.49 to 1.00)		0.85 (0.77 to 0.93)	
**13** Conflicts of interest	0.55 (0.19 to 0.92)		0.52 (0.09 to 0.94)		0.73 (0.44 to 1.00)	
**14** Certainty of findings	0.38 (−0.16 to 0.91)		0.36 (−0.22 to 0.95)		0.63 (0.38 to 0.89)	

Questions marked (∧) had an N/A option. CIs are capped at 1.00. Benchmark interpretation is calculated using 95% cumulative probabilities for Landis and Koch’s benchmark categories, 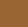
< 0, Poor; 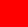
0.0–0.2, Slight; 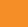
0.2–0.4, Fair; 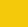
0.4–0.6, Moderate; 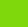
0.6–0.8, Substantial; and 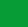
0.8–1.0, Almost Perfect.

AAC, Average Gwet's Agreement Coefficient; AC, Gwet’s agreement coefficient; I, interpretation category; N, no; N/A, not applicable; PF, prognostic factor; PN, partial no; PY, partial yes; RoB, risk of bias; Y, yes.

Agreement scores tended to be higher for the earlier questions in AMSTAR-PF; questions that dealt with review planning, literature searching and study inclusion. Agreement for the latter questions regarding synthesis of results and analysis/interpretation of the included studies generally displayed lower levels of agreement.

### Interrater, inter-pair and intrapair agreement for the overall judgement using AMSTAR-PF

Gwet’s AC2 for 14 appraisers assessing the overall quality of the eight papers was fair 0.46 (95% CI 0.30 to 0.62) and the inter-pair agreement was also fair 0.46 (95% CI 0.17 to 0.74). We found moderate intrapair agreement for overall quality across the seven pairs (average 0.68 (95% CI 0.44 to 0.91)).

Across the 56 intrapair agreements for overall quality, 34 (60.7%) were identical, and a further 19 (33.9%) were one category different. Three decisions (5.4%) differed by two categories.

### Subgroup analysis

Agreement was similar for the first two articles appraised (mean (range of means) intrapair Gwet’s AC=0.76 (0.42–1.00)) and the next six articles (0.74 (0.47–0.95)) (see online supplemental appendix 2, table A, figure A, in the Supplementary material). We decided post hoc to explore potential differences in agreement across the articles further, because we suspected there may be a difference in agreement with the Cochrane (the first and eighth articles appraised) and non-Cochrane articles (see online supplemental appendix 2, table B, figure B, in the supplementary material) because Cochrane articles are generally considered to be more rigorously developed and reported.^29 30^ A difference in the proportion of Cochrane articles within the subgroups, therefore, may have influenced the average agreement and masked changes of agreement as appraisers proceeded. We found that agreement was significantly higher for the two Cochrane articles (mean intrapair AC 0.88 (range of means 0.67–1.00)) than it was for the non-Cochrane articles (0.71 (0.38–0.99)) (p=0.001). Both Cochrane articles were unanimously rated as high quality by every pair; none of the non-Cochrane articles received this level of agreement. Given this higher agreement in Cochrane articles, we performed an additional analysis comparing the first two articles appraised with the final two (because both combinations had one Cochrane and one non-Cochrane article). No statistically significant difference was found in average intrapair agreement (first two articles, (0.76 (range of means 0.42–1.00)); last two articles (0.83 (0.67–1.00)) (p=0.133; (95% CI of difference −0.15 to 0.02)). An additional post hoc analysis was performed to investigate the degree of intrapair difference in the final ratings given to the articles. By coding the final ratings (high, moderate, low, critically low) 1–4, respectively, we were able to assess for differences in the overall ratings given to articles. The first two articles averaged 0.50 categories different; the final two articles averaged 0.21 categories different, but this was not a statistically significant difference (p=0.18).

Across all papers and domains, t-tests for interrater and inter-pair agreement did not suggest a difference between the orders of completion (p=0.59; and p=0.96, respectively); however, the average intrapair agreement did differ between the two orders of completion (p=0.023) (see online supplemental appendix 2, table C, figure C, in the supplementary materials). There was no effect of completion order on how the overall quality of each article was graded (p=0.723; 95% CI of difference −0.36 to 0.51).

### Usability

Time to complete the tool, and time to reach consensus, reduced as appraisers became more familiar with the tool. It took appraisers a median of 55 min (IQR 43–59 min) to complete the AMSTAR-PF appraisal for the first two articles and a median of 33 min (IQR 27–40 min) for the next six articles.

**Figure 4:**
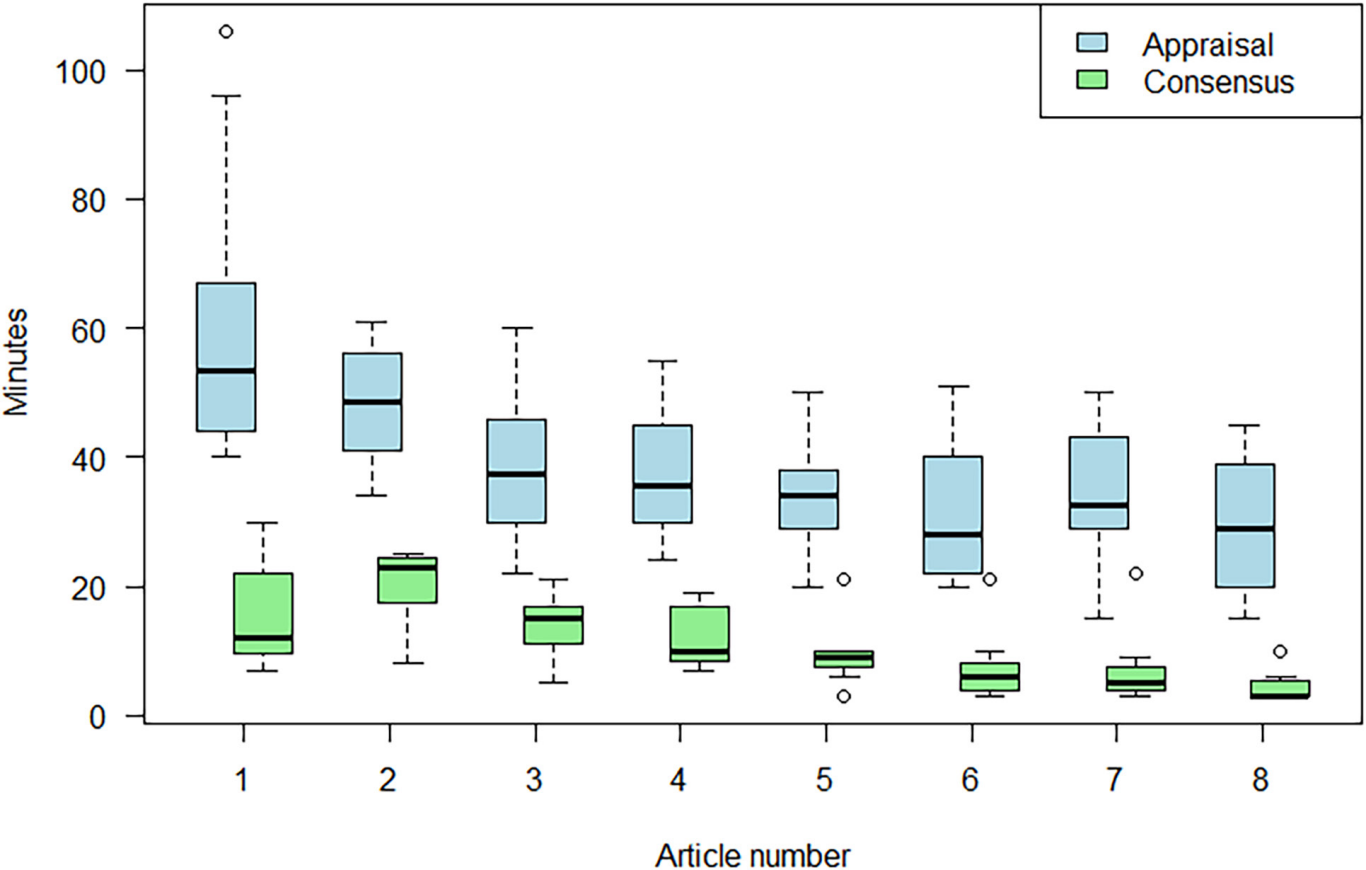
Timings to complete appraisal, and timings to complete consensus, for each article in order of completion. Box represents IQR, horizontal line is the median and the dotted lines the range. Outliers are represented as circles.

It took pairs a median of 18 min (IQR 11–25 min) to reach consensus for the first two articles, and 9 min (IQR 5–12 min) for the final six articles. There were no instances where pairs were unable to reach consensus. See figure 4 and table 3 for more details.

**Table 3:** Timings for appraisal (top table) and consensus (bottom table) for each article in order of completion

Time for appraisal (minutes)							
	**Mean**	**SD**	**Min**	**First quartile**	**Median**	**Third quartile**	**Max**
First article	61	22	40	45	54	65	106
Second article	48	8	34	42	49	55	61
Third article	38	11	22	31	38	45	60
Fourth article	36	9	24	30	36	44	55
Fifth article	35	8	20	29	34	38	50
Sixth article	30	10	20	22	28	38	51
Seventh article	34	10	15	29	33	42	50
Eighth article	30	10	15	21	29	39	45

Time to consensus (minutes)							
	**Mean**	**SD**	**Min**	**First Quartile**	**Median**	**Third Quartile**	**Max**
First article	16	9	7	10	12	22	30
Second article	20	6	8	18	23	25	25
Third article	14	5	5	11	15	17	20
Fourth article	12	5	7	9	10	17	19
Fifth article	10	6	3	8	9	10	21
Sixth article	8	6	3	4	6	8	21
Seventh article	8	7	3	4	5	8	22
Eighth article	5	3	3	3	3	6	10

Completion times were not different between the seven more experienced researchers (5+ years in academic research) (median completion 39 min (IQR 30–51)) and the seven more novice researchers (three or fewer years in research) (median completion 45 min (IQR 30–61)) (p=0.76, 95% CI for difference in completion times −4.0 to 5.0).

### Modification

Some questions were highlighted, either from feedback or the agreement scores, as being more difficult to answer. Some of these were more complicated, or subjective, questions, for example, *whether adequate investigation of small study effects occurred*, or *was the level of certainty around key findings described*? Other questions were more straightforward, and the poor agreement was seen as resulting from a lack of adequate guidance. Examples of this include question 2b: *does the review justify any deviations from the protocol?* Part of the reason for this may have been subjectivity in what constituted *justification* of deviations, but partly also variation in how people responded if there was no protocol, or no publicly available protocol. Specific recommendations were added to the guidance notes to standardise this. Similarly, Q13, *does the review report any potential sources of conflict of interest, both in the individual studies included in the review and among the review author team, including any funding received?* Received low agreement scores, despite the signalling points showing high agreement. In our sample, it was common for articles to report conflicts and funding from the review authors, but not the included studies, and there was a high level of concordance with these being identified by appraisers. Some appraisers, however, routinely marked this situation as PY, others as PN, and some varied between PY and PN without any obvious rationale. More detail was included in the guidance notes to standardise responses in this situation. Similar modifications were made in other questions to try to provide further clarity or more specific guidance; however, the overarching theme of all questions remained the same, and overall changes were relatively minor. Online supplemental appendix 3 in the supplementary material compares the wording of the questions in the tested and final versions of AMSTAR-PF.

## Discussion

We aimed to determine agreement and usability of AMSTAR-PF using (1) measures of interrater, inter-pair and intrapair agreement, and (2) time to complete appraisal using the tool by researchers with a diverse range of research experience. Interrater agreement averaged 0.59 (range 0.21–0.90), inter-pair agreement 0.61 (range 0.24–0.91) and intrapair agreement 0.75 (range 0.45–0.95) across the domains, with agreement for the overall rating 0.46 (95% CI 0.30 to 0.62) for interrater agreement, 0.46 (95% CI 0.17 to 0.74) for inter-pair agreement and 0.68 (range of averages 0.22–1.00) for intrapair agreement. When benchmarked, the agreement scores ranged from slight to almost perfect. Where agreement was lacking, changes and additions were made to the guidance notes of the tool in order to provide further clarity and improve agreement, and the wording of some questions was altered slightly in response to feedback. The time taken to appraise a study with AMSTAR-PF averaged around 34 min, and time to reach consensus was 9.5 min, after the first two appraisals.

The measures of agreement we calculated for AMSTAR-PF appear comparable to measures from other established tools and better than some. Direct comparison of agreement scores is difficult, however, due to a range of measures and weightings used, and differences in testing protocols, the papers assessed and appraisers. Published ranges for Gwet’s AC for ROBIS (Risk of Bias In Systematic reviews)^31^ and AMSTAR 2,^6^ for instance, range between 0.05 and 1.00 for interrater (between two, three or four raters) agreement,^32^,^34^ and −0.21 and 0.74 for inter-pair.^32^ AC statistics for intrapair and inter-pair agreement for the overall risk of bias using ROBINS-I (Risk Of Bias In Non-randomised Studies - of Interventions)^35^ were 0.00 and 0.07, and using ROB-NRSE (Risk Of Bias for Non-Randomised Studies of Exposures) 0.11 and 0.00, respectively.^36^ Published benchmarked agreements when using kappa range from no agreement to moderate agreement for a range of tools, including AMSTAR 2,^33 37 38^ ROBIS,^3337^,^39^ ROB 2 (Revised Cochrane Risk of Bias tool for randomised trials),^40^,^42^ ROBINS-I^43^ and PROBAST (Prediction model Risk Of Bias ASsessment Tool).^44 45^ As illustrated here, there is significant variability in published agreement scores for a range of tools, and the agreement is generally modest. The variance in scores may be a result of different teams of appraisers, papers and interpretations, among other variables. The low agreement scores highlight the difficulties and subjectivity inherent to appraising articles. There is unlikely to be an easy or obvious replacement to quality appraisal tools. Increased use of reporting guidelines is one area that is likely to improve the agreement seen on quality appraisal tools—having a clear indication of what was performed (and how) decreases the subjectivity of appraisers in making their judgements, and although this will not always improve the quality of the review, it can allow for more harmonious judgements by appraisers.

Although both the Gwet’s AC and kappa values were comparable to other tools, it is worth noting differences between the two. Gwet’s AC values were generally higher than the kappa values. This is a common finding^20 25 46^ because kappa is reported to overestimate chance agreement.^16 47^ The well-recognised kappa paradox^17 18^ occurred in questions where agreement was high, with low kappa values leading to large differences noted between Gwet’s AC and kappa. Question 7a illustrates this paradox and the more appropriate values of Gwet’s AC. With all answering options, the percentage of agreement across the 14 appraisers is 87.5%, Gwet’s AC is 0.82 (‘substantial’ agreement), yet Fleiss’ kappa is 0.12 (‘slight’ agreement). The percentage agreement is 87.7% across the seven pairs’ consensus scores, with Gwet’s AC 0.82 (‘substantial’) and Fleiss’ kappa 0.10 (‘slight’). The results when comparing the collapsed answering options are even more extreme; 96.4% agreement (110/112 individual scores agreed, 55/56 consensus scores agreed), Gwet’s AC 0.96 (‘almost perfect’ agreement), and Fleiss’ kappa −0.02 (‘poor’ agreement—with the negative value suggesting agreement worse than chance). In situations where agreement was lower, for example, the agreement scores of the overall rating, Gwet’s AC and kappa are more closely aligned (interrater 0.46 vs 0.46, inter-pair 0.46 vs 0.47 and intrapair 0.68 vs 0.65, for Gwet’s AC vs kappa, respectively). The differences in behaviour of the agreement scores with different prevalences and familiarity of different readers with different agreement measures may point to a benefit in presenting multiple measures to allow for a nuanced picture of agreement levels and facilitate comparability across studies.^48^

The time taken to complete an appraisal using AMSTAR-PF was also comparable to other tools. For example, documented time to complete appraisal tools includes 19–35 min^32^ for AMSTAR 2, 24–28 min for ROBIS,^32^ 28–168 min for ROB 2,^41 42 49^ 27 min^43^ and 48 min^36^ for ROBINS-I and 37 min for ROB-NRSE.^36^ While different articles, appraisers and tools will contribute to variability in completion times, some of the difference may also be explained by variations in the protocol used. Some studies (eg, studies by Gates *et al* and Lee^32 38^) used a protocol similar to ours, whereby only the time taken using the tool was recorded. Other studies (eg, studies by Jeyaraman *et al*, Minozzi *et al* and Minozzi *et al*^36 41 43^) included the time taken to read the review, which we did not. We decided against this because we considered it removed the impact of the length of article and appraisers’ reading speeds on our results. Our focus was on the time to use the tool, not the complete time burden of appraising articles more generally.

Our testing showed that intrapair agreement tended to be stronger than inter-pair or interrater agreement. This may point to a benefit in ensuring teams take time to discuss different aspects of the papers under review, and the relation to their field or review question, in order to better standardise responses and align interpretation. In our testing procedure, this occurred after two papers had been appraised, but there may be benefit in also meeting prior to appraising any articles if potential issues are already known or envisioned beforehand. Guides such as the Cochrane handbook recommend planning and piloting appraisals.^15^

Familiarity with the tool (and perhaps PF research and systematic reviews more generally) may reduce time to complete appraisals and gain consensus. Usability data showed improved timing as people progressed through the allocated papers, irrespective of the order of completion. Interestingly, there was no significant change in agreement scores over the course of the testing. This suggests that learning effects may occur in efficiency, but have little impact on how studies are scored. The exploratory comparison of the Cochrane versus non-Cochrane articles found that Cochrane articles showed higher levels of agreement. This is unsurprising, as Cochrane articles are reported to have higher standards of reporting, which may make answering questions easier as the information is more explicit. Appraisers were not, however, blinded to the publication details, so may have also assumed the information they were seeking was recorded and more consistently and thoroughly looked for it. Future testing of appraisal tools may consider removing details such as journal name, author details and institutions, to allow for blinded appraisal and prespecifying this as an outcome of interest.

To our knowledge, AMSTAR-PF is the only tool specifically designed for reviews of PF studies, but other more general tools have been used. Most published systematic reviews undertake some form of quality assessment,^50^ but these vary markedly; one review found that 54 combinations of assessment tools were used across a sample of 309 reviews.^50^ While there are similarities in aims and constructs in many of these tools, previous research has shown that the use of different tools may lead to differing and indeed sometimes opposite conclusions of quality.^51^

Strengths of this study include: the lodging of a full protocol prior to data collection and clearly stipulating when we deviated from that protocol; the number of appraisers involved; the diversity of experience levels; and the diversity in review topics. Articles were standardised across all appraisers to ensure a broader understanding of inter-pair agreement, and the counterbalanced order of completion helped to ensure there were direct comparisons among pairs who completed the same order, while also allowing comparison to detect an order effect on appraisals. Appraisers convened in the early stages of the review, which is a recommended method when applying appraisal tools in research.^15^

A potential limitation to this testing protocol was the lack of PF experts in the appraiser group. Including methodological experts may have added more information and a valuable comparison to the results presented in this paper, and may be a topic for further research. We considered, however, that in practice it is often research students and early career researchers who do the bulk of the quality appraisal component of systematic reviews and umbrella reviews, although often with expert/s in the team they can consult when needed. Similarly, the use of articles outside the appraisers’ topic knowledge may be considered a limitation. We consider, however, that this approach has ecological validity because it is common for junior researchers to be involved in quality appraisal for reviews, which often will be on topics outside their expertise. Furthermore, we considered that if appraisers could appropriately use the tool on reviews of unfamiliar topics, they should be at least as comfortable and efficient on reviews in more familiar subject areas. All appraisers had a Cochrane review as their initial article to appraise. We acknowledge that in practice, this will not always be the case, but we believe that the benefit of including Cochrane and non-Cochrane reviews, and being able to compare agreement in a standardised order, outweighed this limitation. Using Landis and Koch’s benchmarks for Gwet’s AC scores is a potential limitation and has been criticised (eg, by Vach and Gerke^46^); however, they are commonly used to help interpret Gwet’s ACs, in part due to a lack of clear alternatives. We chose this approach to provide an interpretation of the agreement scores, but mitigated the potential limitation by using SE to calculate cumulative probabilities, as recommended by Gwet,^23 27^ which give more conservative interpretations of the benchmarks than simply reporting where on Landis and Koch’s scale the AC score lies. We have also calculated Fleiss’ and Cohen’s kappa and benchmarked these as a comparison (online supplemental appendix 4 in the supplementary material). Verbal rating scales as a broader concept have been criticised as being subjective and field-dependent or situation-dependent,^52^ but remain common practice as a means of trying to convey meaning about coefficient values. The limitations associated with them are worth considering, as are the potential negatives of not using any form of interpretation. It is possible that including more articles for appraisal may have further improved efficiency or agreement, but we arbitrarily chose eight as a number that would provide a good indication of change in efficiency or agreement with time, allow an assortment of different article topics and analyses and be manageable for appraisers. Finally, we did not contact authors of the included reviews in situations where reporting was unclear or additional data may have been beneficial in coming to a judgement; meaning there were potentially more areas of uncertainty than there would be if a complete umbrella review was being performed, as opposed to just quality appraisal. That is, adding this step in practice may improve agreement and thus performance of the AMSTAR-PF tool.

## Conclusion

AMSTAR-PF displays an acceptable level of usability and agreement, offering a valuable tool for those appraising the quality of systematic reviews of PFs. There may be benefit for appraiser pairs to meet prior to commencing quality appraisal, to ensure standardised interpretation of the tool and how the different domains may be relevant to the topic area of the reviews under appraisal.

## Supplementary material

10.1136/bmjopen-2025-109388 online supplemental file 1

## Data Availability

Data are available upon reasonable request.
