# Supplementary material for: Agreement testing of AMSTAR-PF, a tool for quality appraisal of systematic reviews of prognostic factor studies
Source: BMJ Open. 2026 Jan 27;16(1):e109388. doi: 10.1136/bmjopen-2025-109388 (PMC12853518; doi:10.1136/bmjopen-2025-109388)
Supplement: online supplemental file 1 [file bmjopen-16-1-s001.docx]

**Supplementary Material**

**Appendix I – AMSTAR-PF**

| **1** | Did the review clearly define the research question, including the relevant components of PICOTS? |
| --- | --- |
| **2a** | Did the review state that a protocol was registered prior to the conduct of the review, and is that registration publicly available? |
| **2b** | Did the review justify any deviations from the protocol? |
| **3** | Did the review report the types of prognostic factor studies eligible for inclusion in the review, eg, which primary study designs were eligible? |
| **4** | Did the review use a comprehensive search strategy to identify relevant studies? |
| **5** | Was a rigorous process followed when evaluating studies for inclusion into the review? |
| **6** | Did the review list all excluded studies that were included in the full text screening, and the reasons for those exclusions? |
| **7a** | Was a rigorous process followed when performing data extraction for each included study? |
| **7b** | Did the review describe the included studies in adequate detail? |
| **7c** | Did the review use appropriate techniques for calculating or obtaining the Prognostic Factor effect estimates and their precision, in situations they were not fully reported in the primary studies? |
| **8a** | Was a rigorous process followed when performing risk of bias (RoB) assessment for each included study? |
| **8b** | Did the review use an appropriate technique for assessing the risk of bias (RoB) of individual studies that were included in the review? |
| **9a** | If synthesis was performed, did the approach taken ensure the interpretability of results? |
| **9b** | If meta-analysis was performed, did the review use appropriate analysis methods? |
| **10** | If the review performed quantitative data synthesis, did it also carry out an adequate investigation of small study effects? |
| **11** | Did the review account for risk of bias (RoB) in individual studies when interpreting/discussing the results of the review? |
| **12** | Did the review discuss any heterogeneity observed in its results? |
| **13** | Did the review report any potential sources of conflict of interest, both in the individual studies included in the review and among the review author team, including any funding received? |
| **14** | Did the review address the level of certainty around their key findings and use appropriate methods to come to an overall certainty judgement for each outcome? |

**Summary of AMSTAR-PF questions** (from Henry ML, O’Connell NE, Riley RD, et al. AMSTAR-PF: a critical appraisal tool for systematic reviews of prognostic factor studies. *BMJ*, 2025; 391:e085718 doi:10.1136/bmj-2025-085718). Note that the testing used a slightly different version of the tool; see Appendix III in these supplementary files for a comparison of the AMSTAR-PF version used for testing compared to the published version shown here.

**Appendix II – Subgroup comparisons of article agreement**

|  | First two articles | | Last six articles | | Last two articles | |
| --- | --- | --- | --- | --- | --- | --- |
| Question | **AC** | **SEM** | **AC** | **SEM** | **AC** | **SEM** |
| 1 | 1.00 | 0.00 | 0.80 | 0.06 | 0.85 | 0.10 |
| 2a | 0.83 | 0.09 | 0.92 | 0.04 | 0.76 | 0.13 |
| 2b | 0.49 | 0.16 | 0.55 | 0.07 | 0.67 | 0.12 |
| 3 | 0.97 | 0.03 | 0.78 | 0.09 | 0.83 | 0.10 |
| 4 | 0.74 | 0.11 | 0.83 | 0.04 | 0.93 | 0.04 |
| 5 | 0.87 | 0.09 | 0.82 | 0.06 | 0.87 | 0.07 |
| 6 | 0.69 | 0.13 | 0.88 | 0.06 | 0.92 | 0.05 |
| 7a | 0.87 | 0.10 | 0.94 | 0.04 | 0.97 | 0.03 |
| 7b | 0.84 | 0.09 | 0.81 | 0.04 | 0.82 | 0.07 |
| 7c | 0.42 | 0.18 | 0.47 | 0.08 | 0.67 | 0.21 |
| 8a | 0.78 | 0.14 | 0.92 | 0.04 | 0.97 | 0.03 |
| 8b | 0.93 | 0.07 | 0.95 | 0.02 | 1.00 | 0.00 |
| 9a | 0.84 | 0.10 | 0.54 | 0.12 | 0.67 | 0.17 |
| 9b | 0.52 | 0.15 | 0.80 | 0.04 | 0.76 | 0.11 |
| 10 | 0.75 | 0.17 | 0.54 | 0.12 | 0.75 | 0.12 |
| 11 | 0.87 | 0.06 | 0.61 | 0.13 | 0.85 | 0.07 |
| 12 | 0.67 | 0.17 | 0.59 | 0.05 | 0.68 | 0.11 |
| 13 | 0.74 | 0.10 | 0.81 | 0.07 | 0.95 | 0.05 |
| 14 | 0.81 | 0.10 | 0.52 | 0.10 | 0.77 | 0.10 |
| Final | 0.63 | 0.12 | 0.70 | 0.11 | 0.87 | 0.06 |
|  |  |  |  |  |  |  |
| Mean | **0.76** |  | **0.74** |  | **0.83** |  |
| SEM | 0.03 |  | 0.04 |  | 0.02 |  |
| SD | 0.16 |  | 0.16 |  | 0.11 |  |
| min | 0.42 |  | 0.47 |  | 0.67 |  |
| max | 1.00 |  | 0.95 |  | 1.00 |  |
| Benchmark | 0.6-0.8 |  | 0.6-0.8 |  | 0.6-0.8 |  |

**Table A:** **Average Gwet’s AC for the intrapair agreement for the first two, final six, and final two articles appraised**. Benchmark interpretation is calculated using 95% cumulative probabilities for Landis and Koch’s benchmark categories:

⬛< 0, Poor; ⬛ 0.0-0.2, Slight; ⬛0.2-0.4, Fair; ⬛0.4-0.6, Moderate; ⬛0.6-0.8, Substantial; and ⬛0.8-1.0, Almost Perfect.

AC, Gwet’s Agreement Coefficient; min, minimum; max, maximum; SEM, Standard Error of the Mean; SD, Standard Deviation

**Figure A:** **Average intrapair Gwet’s AC for the first two, last six, and last two articles appraised.** Error bars are standard error of the mean.

|  | Interrater | | | | Inter-pair | | | | Intrapair | | | |
| --- | --- | --- | --- | --- | --- | --- | --- | --- | --- | --- | --- | --- |
|  | **Cochrane** | | **Non-Cochrane** | | **Cochrane** | | **Non-Cochrane** | | **Cochrane** | | **Non-Cochrane** | |
| Question | **AC** | **SE** | **AC** | **SE** | **AC** | **SE** | **AC** | **SE** | **Avg AC** | **SEM** | **Avg AC** | **SEM** |
| 1 | 0.97 | 0.03 | 0.57 | 0.11 | 0.95 | 0.06 | 0.57 | 0.10 | 0.97 | 0.03 | 0.79 | 0.07 |
| 2a | 0.39 | 0.27 | 0.99 | 0.01 | 0.20 | 0.17 | 1.00 | 0.00 | 0.67 | 0.14 | 0.99 | 0.01 |
| 2b | 0.85 | 0.00 | 0.13 | 0.04 | 0.70 | 0.00 | 0.17 | 0.05 | 0.84 | 0.10 | 0.45 | 0.10 |
| 3 | 1.00 | 0.00 | 0.70 | 0.14 | 1.00 | 0.00 | 0.83 | 0.12 | 1.00 | 0.00 | 0.77 | 0.10 |
| 4 | 0.87 | 0.08 | 0.67 | 0.04 | 0.77 | 0.00 | 0.76 | 0.06 | 0.90 | 0.05 | 0.76 | 0.08 |
| 5 | 0.92 | 0.08 | 0.79 | 0.10 | 0.90 | 0.12 | 0.81 | 0.13 | 0.90 | 0.07 | 0.81 | 0.07 |
| 6 | 0.81 | 0.15 | 0.81 | 0.05 | 0.76 | 0.14 | 0.91 | 0.02 | 0.89 | 0.05 | 0.86 | 0.06 |
| 7a | 0.91 | 0.11 | 0.79 | 0.07 | 0.90 | 0.12 | 0.80 | 0.08 | 0.93 | 0.04 | 0.91 | 0.04 |
| 7b | 0.97 | 0.03 | 0.62 | 0.09 | 0.95 | 0.06 | 0.66 | 0.05 | 0.97 | 0.03 | 0.76 | 0.08 |
| 7c | 0.45 | 0.16 | 0.14 | 0.06 | 0.47 | 0.24 | 0.17 | 0.05 | 0.67 | 0.17 | 0.38 | 0.07 |
| 8a | 0.86 | 0.10 | 0.78 | 0.12 | 0.87 | 0.16 | 0.81 | 0.12 | 0.93 | 0.04 | 0.88 | 0.06 |
| 8b | 1.00 | 0.00 | 0.86 | 0.11 | 1.00 | 0.00 | 0.87 | 0.12 | 1.00 | 0.00 | 0.93 | 0.03 |
| 9a | 0.81 | 0.20 | 0.28 | 0.11 | 0.75 | 0.27 | 0.23 | 0.10 | 0.92 | 0.08 | 0.51 | 0.13 |
| 9b | 0.85 | 0.00 | 0.51 | 0.12 | 1.00 | 0.00 | 0.60 | 0.09 | 0.84 | 0.10 | 0.69 | 0.07 |
| 10 | 0.55 | 0.32 | 0.16 | 0.11 | 0.60 | 0.44 | 0.15 | 0.11 | 0.76 | 0.11 | 0.54 | 0.11 |
| 11 | 0.95 | 0.00 | 0.09 | 0.10 | 1.00 | 0.00 | 0.13 | 0.06 | 0.93 | 0.04 | 0.59 | 0.13 |
| 12 | 0.52 | 0.08 | 0.24 | 0.12 | 0.47 | 0.00 | 0.33 | 0.11 | 0.68 | 0.11 | 0.57 | 0.09 |
| 13 | 0.97 | 0.03 | 0.50 | 0.10 | 1.00 | 0.00 | 0.39 | 0.07 | 0.97 | 0.03 | 0.78 | 0.06 |
| 14 | 1.00 | 0.00 | 0.10 | 0.09 | 1.00 | 0.00 | 0.14 | 0.14 | 1.00 | 0.00 | 0.45 | 0.10 |
| Final | 0.89 | 0.00 | 0.44 | 0.06 | 1.00 | 0.00 | 0.38 | 0.06 | 0.85 | 0.08 | 0.70 | 0.09 |
|  |  |  |  |  |  |  |  |  |  |  |  |  |
| Mean | **0.83** |  | **0.51** |  | **0.81** |  | **0.54** |  | **0.88** |  | **0.71** |  |
| SEM | 0.04 |  | 0.07 |  | 0.05 |  | 0.07 |  | 0.02 |  | 0.04 |  |
| SD | 0.19 |  | 0.29 |  | 0.23 |  | 0.30 |  | 0.11 |  | 0.18 |  |
| min | 0.39 |  | 0.09 |  | 0.20 |  | 0.13 |  | 0.67 |  | 0.38 |  |
| max | 1.00 |  | 0.99 |  | 1.00 |  | 1.00 |  | 1.00 |  | 0.99 |  |
| B-M | 0.6-0.8 |  | 0.4-0.6 |  | 0.6-0.8 |  | 0.4-0.6 |  | 0.8-1.0 |  | 0.6-0.8 |  |

**Table B: Gwet’s AC for Interrater, Inter-pair, and Intrapair agreement for the Cochrane and non-Cochrane articles.** Benchmark interpretation is calculated using 95% cumulative probabilities for Landis and Koch’s benchmark categories:

⬛< 0, Poor; ⬛ 0.0-0.2, Slight; ⬛0.2-0.4, Fair; ⬛0.4-0.6, Moderate; ⬛0.6-0.8, Substantial; and ⬛0.8-1.0, Almost Perfect.

AC, Gwet’s Agreement Coefficient; Avg, Average; B-M, Benchmark interpretation; min, minimum; max, maximum; SD, Standard Deviation; SE, Standard Error; SEM, Standard Error of the Mean.

**Figure B: Average intrapair Gwet’s AC for the Cochrane and non-Cochrane articles**. Error bars are standard error of the mean.

|  | Interrater | | | | Inter-pair | | | | Intrapair | | | |
| --- | --- | --- | --- | --- | --- | --- | --- | --- | --- | --- | --- | --- |
|  | **Stream 1** | | **Stream 2** | | **Stream 1** | | **Stream 2** | | **Stream 1** | | **Stream 2** | |
| Question | **AC** | **SE** | **AC** | **SE** | **AC** | **SE** | **AC** | **SE** | **Avg AC** | **SEM** | **Avg AC** | **SEM** |
| 1 | 0.68 | 0.08 | 0.75 | 0.12 | 0.71 | 0.07 | 0.71 | 0.13 | 0.83 | 0.06 | 0.84 | 0.09 |
| 2a | 0.88 | 0.11 | 0.85 | 0.10 | 0.84 | 0.13 | 0.83 | 0.14 | 0.98 | 0.02 | 0.84 | 0.07 |
| 2b | 0.32 | 0.12 | 0.25 | 0.15 | 0.23 | 0.12 | 0.25 | 0.12 | 0.55 | 0.09 | 0.51 | 0.11 |
| 3 | 0.85 | 0.06 | 0.77 | 0.14 | 0.88 | 0.08 | 0.93 | 0.08 | 0.95 | 0.05 | 0.75 | 0.09 |
| 4 | 0.77 | 0.07 | 0.65 | 0.09 | 0.83 | 0.07 | 0.69 | 0.02 | 0.86 | 0.04 | 0.75 | 0.07 |
| 5 | 0.87 | 0.07 | 0.80 | 0.10 | 0.88 | 0.08 | 0.79 | 0.12 | 0.88 | 0.06 | 0.80 | 0.07 |
| 6 | 0.69 | 0.12 | 0.78 | 0.05 | 0.64 | 0.14 | 0.94 | 0.05 | 0.88 | 0.07 | 0.79 | 0.11 |
| 7a | 0.85 | 0.06 | 0.80 | 0.08 | 0.84 | 0.08 | 0.81 | 0.10 | 0.95 | 0.01 | 0.89 | 0.06 |
| 7b | 0.77 | 0.09 | 0.63 | 0.11 | 0.75 | 0.07 | 0.59 | 0.09 | 0.90 | 0.02 | 0.73 | 0.07 |
| 7c | 0.12 | 0.04 | 0.30 | 0.12 | 0.09 | 0.07 | 0.37 | 0.12 | 0.51 | 0.13 | 0.40 | 0.14 |
| 8a | 0.80 | 0.07 | 0.80 | 0.11 | 0.79 | 0.08 | 0.83 | 0.12 | 0.93 | 0.07 | 0.87 | 0.05 |
| 8b | 0.91 | 0.07 | 0.89 | 0.09 | 0.92 | 0.08 | 0.93 | 0.08 | 0.98 | 0.02 | 0.93 | 0.03 |
| 9a | 0.48 | 0.11 | 0.43 | 0.13 | 0.41 | 0.12 | 0.29 | 0.13 | 0.70 | 0.09 | 0.55 | 0.19 |
| 9b | 0.68 | 0.10 | 0.64 | 0.13 | 0.80 | 0.13 | 0.66 | 0.14 | 0.70 | 0.09 | 0.75 | 0.09 |
| 10 | 0.24 | 0.14 | 0.28 | 0.10 | 0.28 | 0.17 | 0.28 | 0.14 | 0.55 | 0.15 | 0.62 | 0.05 |
| 11 | 0.43 | 0.13 | 0.42 | 0.17 | 0.36 | 0.19 | 0.42 | 0.21 | 0.89 | 0.03 | 0.54 | 0.11 |
| 12 | 0.49 | 0.14 | 0.44 | 0.14 | 0.41 | 0.14 | 0.48 | 0.14 | 0.71 | 0.00 | 0.52 | 0.08 |
| 13 | 0.70 | 0.12 | 0.50 | 0.13 | 0.47 | 0.19 | 0.55 | 0.15 | 0.87 | 0.07 | 0.73 | 0.07 |
| 14 | 0.44 | 0.19 | 0.37 | 0.23 | 0.43 | 0.19 | 0.42 | 0.25 | 0.61 | 0.15 | 0.58 | 0.08 |
| Final | 0.58 | 0.09 | 0.45 | 0.08 | 0.57 | 0.12 | 0.44 | 0.14 | 0.89 | 0.06 | 0.52 | 0.10 |
|  |  |  |  |  |  |  |  |  |  |  |  |  |
| Mean | **0.63** |  | **0.59** |  | **0.61** |  | **0.61** |  | **0.81** |  | **0.70** |  |
| SEM | 0.05 |  | 0.05 |  | 0.06 |  | 0.05 |  | 0.03 |  | 0.03 |  |
| SD | 0.23 |  | 0.21 |  | 0.25 |  | 0.23 |  | 0.15 |  | 0.15 |  |
| min | 0.12 |  | 0.25 |  | 0.09 |  | 0.25 |  | 0.51 |  | 0.40 |  |
| max | 0.91 |  | 0.89 |  | 0.92 |  | 0.94 |  | 0.98 |  | 0.93 |  |
| B-M | 0.4-0.6 |  | 0.4-0.6 |  | 0.4-0.6 |  | 0.4-0.6 |  | 0.6-0.8 |  | 0.6-0.8 |  |

**Table C: Gwet’s AC for Interrater, Inter-pair, and Intrapair agreement for the two different orders of completion.** Benchmark interpretation is calculated using 95% cumulative probabilities for Landis and Koch’s benchmark categories:

⬛< 0, Poor; ⬛ 0.0-0.2, Slight; ⬛0.2-0.4, Fair; ⬛0.4-0.6, Moderate; ⬛0.6-0.8, Substantial; and ⬛0.8-1.0, Almost Perfect.

AC, Gwet’s Agreement Coefficient; Avg, Average; B-M, Benchmark; min, minimum; max, maximum; SD, Standard Deviation; SE, Standard Error; SEM, Standard Error of the Mean

**Figure C:** **Average interrater, inter-pair, and intrapair Gwet’s AC for two orders of completion**. Error bars are standard error of the mean. *Signifies significant difference at p < 0.005

**Appendix III – AMSTAR-PF – Tested version compared to the final version**

| **1** | ~~Does~~ Did the review clearly define the research question, including the relevant components of PICOTS? |
| --- | --- |
| **2a** | ~~Does~~ Did the review ~~contain a clear statement~~ state that a protocol was registered prior to the conduct of the review, and is that ~~the~~ registration ~~is~~ publicly available? |
| **2b** | ~~Does~~ Did the review justify any deviations from the protocol? |
| **3** | ~~Does~~ Did the review report the types of prognostic factor studies eligible for inclusion in the review, eg, which primary study designs were eligible? |
| **4** | ~~Does~~ Did the review use a comprehensive search strategy to identify relevant studies? |
| **5** | Was a rigorous process followed when evaluating studies for inclusion into the review? |
| **6** | ~~Does~~ Did the review list all excluded studies that were included in the full text screening, and the reasons for those exclusions? |
| **7a** | Was a rigorous process followed when performing data extraction for each included study? |
| **7b** | ~~Does~~ Did the review describe the included studies in adequate detail? |
| **7c** | ~~Does~~ Did the review use appropriate techniques for calculating or obtaining the Prognostic Factor effect estimates and their precision, in situations they were not fully reported in the primary studies? |
| **8a** | Was a rigorous process followed when performing risk of bias (RoB) assessment for each included study? |
| **8b** | ~~Does~~ Did the review use an appropriate technique for assessing the risk of bias (RoB) of individual studies that were included in the review? |
| **9a** | If ~~data~~ synthesis was performed, ~~does~~ did the approach taken ensure the interpretability of results? |
| **9b** | If meta-analysis was performed, ~~does~~ did the review use appropriate analysis methods? |
| **10** | If the review performed quantitative data synthesis, ~~does~~ did it also carry out an adequate investigation of small study effects? |
| **11** | ~~Does~~ Did the review account for risk of bias (RoB) in individual studies when interpreting/discussing the results of the review? |
| **12** | ~~Does~~ Did the review discuss any heterogeneity observed in its results? |
| **13** | ~~Does~~ Did the review report any potential sources of conflict of interest, both in the individual studies included in the review and among the review author team, including any funding received? |
| **14** | ~~Does~~ Did the review address the level of certainty around their key findings and use appropriate methods to come to an overall certainty judgement for each outcome? |

**Summary of AMSTAR-PF questions in the final version of the tool, compared with the version tested.** Words in blue font have been added for the final version; words with strikethrough have been removed.

**Appendix IV – Kappa scores for agreement**

| **Question** | **Interrater** | | | | **Inter-pair** | | | | **Intrapair** | | | |
| --- | --- | --- | --- | --- | --- | --- | --- | --- | --- | --- | --- | --- |
|  | **F Kap** | **95% CI** | | I | **F Kap** | **95% CI** | | I | **C Kap** | **95% CI** | | I |
| **1** Research Question | 0.27 | 0.06 | 0.49 |  | 0.21 | -0.04 | 0.46 |  | 0.64 | 0.42 | 0.85 |  |
| **2a** Protocol Registration | 0.74 | 0.29 | 1.00 |  | 0.69 | 0.19 | 1.00 |  | 0.83 | 0.67 | 0.98 |  |
| **^2b** Deviations from protocol | 0.27 | 0.01 | 0.53 |  | 0.25 | 0.04 | 0.47 |  | 0.44 | 0.24 | 0.63 |  |
| **3** Included study designs | 0.12 | 0.00 | 0.25 |  | 0.27 | 0.05 | 0.49 |  | 0.42 | -0.11 | 0.94 |  |
| **4** Search strategy | 0.23 | 0.04 | 0.42 |  | 0.14 | -0.05 | 0.32 |  | 0.40 | 0.11 | 0.68 |  |
| **5** Inclusion process | 0.20 | -0.02 | 0.41 |  | 0.35 | 0.12 | 0.58 |  | 0.40 | 0.06 | 0.74 |  |
| **6** Excluded studies | 0.65 | 0.37 | 0.94 |  | 0.72 | 0.53 | 0.92 |  | 0.76 | 0.57 | 0.95 |  |
| **7a** Data extraction | 0.12 | -0.05 | 0.30 |  | 0.10 | -0.08 | 0.28 |  | 0.52 | 0.13 | 0.91 |  |
| **7b** Description of studies | 0.34 | 0.06 | 0.62 |  | 0.39 | 0.09 | 0.68 |  | 0.57 | 0.37 | 0.77 |  |
| **^7c** PF effect calculations | 0.12 | -0.01 | 0.25 |  | 0.11 | -0.10 | 0.33 |  | 0.32 | 0.13 | 0.50 |  |
| **8a** RoB process | 0.27 | 0.02 | 0.51 |  | 0.34 | 0.06 | 0.63 |  | 0.71 | 0.41 | 1.00 |  |
| **8b** RoB technique | 0.35 | 0.16 | 0.53 |  | 0.35 | 0.20 | 0.50 |  | 0.59 | 0.15 | 1.00 |  |
| **^9a** Synthesis interpretability | 0.31 | -0.02 | 0.64 |  | 0.24 | -0.11 | 0.59 |  | 0.54 | 0.27 | 0.81 |  |
| **^9b** Meta-Analysis | 0.44 | 0.08 | 0.80 |  | 0.57 | 0.24 | 0.91 |  | 0.62 | 0.42 | 0.82 |  |
| **^10** Small study effects | 0.22 | -0.05 | 0.49 |  | 0.21 | -0.12 | 0.55 |  | 0.53 | 0.39 | 0.67 |  |
| **11** Impact of RoB | 0.25 | -0.03 | 0.54 |  | 0.27 | -0.07 | 0.62 |  | 0.59 | 0.32 | 0.87 |  |
| **^12** Heterogeneity | 0.16 | 0.01 | 0.31 |  | 0.21 | 0.06 | 0.36 |  | 0.45 | 0.31 | 0.60 |  |
| **13** Conflicts of interest | 0.30 | -0.03 | 0.64 |  | 0.24 | -0.11 | 0.59 |  | 0.48 | 0.16 | 0.80 |  |
| **14** Certainty of findings | 0.34 | 0.06 | 0.62 |  | 0.38 | 0.05 | 0.71 |  | 0.46 | 0.27 | 0.65 |  |
|  |  | | | |  | | | |  | | | |
| **Final Appraisal** | **0.45** | **0.10** | **0.80** |  | **0.47** | **0.04** | **0.90** |  | **0.65** | **0.43** | **0.86** |  |

**Table A: Kappa scores for Interrater, Inter-pair, and Intrapair agreement, with all answering options.** Fleiss’ Kappa was used for Interrater and Inter-pair agreement calculations, and average Cohen’s Kappa is displayed for intrapair agreement. Benchmark interpretation per Landis and Koch’s benchmark categories:

⬛< 0, Poor; ⬛ 0.0-0.2, Slight; ⬛0.2-0.4, Fair; ⬛0.4-0.6, Moderate; ⬛0.6-0.8, Substantial; and ⬛0.8-1.0, Almost Perfect.

C Kap, average Cohen’s Kappa; F Kap, Fleiss’ Kappa; I, interpretation; 95%CI, 95% Confidence Interval.

| **Question** | **Interrater** | | | | **Inter-pair** | | | | **Intrapair** | | | |
| --- | --- | --- | --- | --- | --- | --- | --- | --- | --- | --- | --- | --- |
|  | **F Kap** | **95% CI** | | I | **F Kap** | **95% CI** | | I | **C Kap** | **95% CI** | | I |
| **1** Research Question | 0.27 | -0.02 | 0.56 |  | 0.34 | 0.08 | 0.61 |  | 0.87 | 0.65 | 1.00 |  |
| **2a** Protocol Registration | 0.72 | 0.15 | 1.00 |  | 0.68 | 0.04 | 1.00 |  | 0.85 | 0.62 | 1.00 |  |
| **^2b** Deviations from protocol | 0.34 | -0.01 | 0.69 |  | 0.40 | -0.02 | 0.82 |  | 0.53 | 0.23 | 0.83 |  |
| **3** Included study designs | 0.05 | -0.03 | 0.14 |  | -0.04 | -0.10 | 0.02 |  | 0.54 | 0.01 | 1.07 |  |
| **4** Search strategy | 0.03 | -0.05 | 0.11 |  | -0.04 | -0.10 | 0.02 |  | 0.33 | -0.10 | 0.77 |  |
| **5** Inclusion process | 0.00 | -0.06 | 0.05 |  | 0.14 | 0.06 | 0.21 |  | 0.39 | -0.14 | 0.92 |  |
| **6** Excluded studies | 0.86 | 0.66 | 1.00 |  | 0.90 | 0.71 | 1.00 |  | 0.96 | 0.86 | 1.00 |  |
| **7a** Data extraction | -0.02 | -0.05 | 0.01 |  | -0.02 | -0.06 | 0.03 |  | 0.71 | 0.26 | 1.00 |  |
| **7b** Description of studies | 0.11 | -0.02 | 0.24 |  | 0.11 | -0.08 | 0.30 |  | 0.53 | 0.06 | 1.00 |  |
| **^7c** PF effect calculations | 0.22 | -0.04 | 0.48 |  | 0.21 | -0.13 | 0.55 |  | 0.49 | 0.24 | 0.75 |  |
| **8a** RoB process | 0.36 | 0.21 | 0.51 |  | 0.46 | 0.36 | 0.56 |  | 0.71 | 0.26 | 1.00 |  |
| **8b** RoB technique | 0.21 | 0.09 | 0.34 |  | 0.14 | 0.06 | 0.21 |  | 0.71 | 0.26 | 1.00 |  |
| **^9a** Synthesis interpretability | 0.44 | -0.02 | 0.89 |  | 0.36 | -0.14 | 0.86 |  | 0.63 | 0.35 | 0.91 |  |
| **^9b** Meta-Analysis | 0.72 | 0.37 | 1.00 |  | 0.91 | 0.74 | 1.00 |  | 0.81 | 0.57 | 1.00 |  |
| **^10** Small study effects | 0.30 | -0.02 | 0.62 |  | 0.20 | -0.19 | 0.60 |  | 0.66 | 0.38 | 0.95 |  |
| **11** Impact of RoB | 0.23 | -0.02 | 0.49 |  | 0.18 | -0.07 | 0.43 |  | 0.59 | 0.25 | 0.92 |  |
| **^12** Heterogeneity | 0.43 | 0.23 | 0.63 |  | 0.56 | 0.39 | 0.73 |  | 0.57 | 0.26 | 0.87 |  |
| **13** Conflicts of interest | 0.08 | -0.05 | 0.21 |  | 0.05 | -0.09 | 0.18 |  | 0.51 | 0.04 | 0.98 |  |
| **14** Certainty of findings | 0.26 | 0.00 | 0.52 |  | 0.30 | -0.03 | 0.63 |  | 0.45 | 0.15 | 0.76 |  |

**Table B: Kappa scores for Interrater, Inter-pair, and Intrapair agreement, with collapsed answering options.** Fleiss’ Kappa was used for Interrater and Inter-pair agreement calculations, and average Cohen’s Kappa is displayed for intrapair agreement. Benchmark interpretation per Landis and Koch’s benchmark categories:

⬛< 0, Poor; ⬛ 0.0-0.2, Slight; ⬛0.2-0.4, Fair; ⬛0.4-0.6, Moderate; ⬛0.6-0.8, Substantial; and ⬛0.8-1.0, Almost Perfect.

C Kap, average Cohen’s Kappa; F Kap, Fleiss’ Kappa; I, interpretation; 95%CI, 95% Confidence Interval.
