# Supplementary material for: Agreement testing of AMSTAR-PF, a tool for quality appraisal of systematic reviews of prognostic factor studies
Source: BMJ Open. 2026 Jan 27;16(1):e109388. doi: 10.1136/bmjopen-2025-109388 (PMC12853518; doi:10.1136/bmjopen-2025-109388)
Supplement: Supplementary file 2 [file bmjopen-2025-109388.reviewer_comments.pdf]

PEER REVIEW HISTORY

BMJ Open publishes all reviews undertaken for accepted manuscripts. Reviewers are asked to complete a checklist review form and are provided with free text boxes to elaborate on their assessment. These free text comments are reproduced below.

ARTICLE DETAILS

Title (Provisional)

Agreement testing of AMSTAR-PF, a tool for quality appraisal of systematic reviews of prognostic factor studies

Authors

Henry, Michael Laurence; O'Connell, Neil E; Riley, Richard; Moons, Karel GM; Shea, Beverley; Hooft, Lotty; Wallwork, Sarah; Damen, Johanna A.A.G.; Skoetz, Nicole; Appiah, Ruth; Berryman, Carolyn; Crouch, Sophie; Ferencz, Grace; Grant, Ashley; Henry, Katherine; Herman, Aleksandra; Karran, Emma; Koralegedera, Indika; Leake, Hayley; MacIntyre , Erin; Mouatt, Brendan; Phuentsho, Karma; Van Der Laan, Daniel; Welsby, Ellana; Wiles, Louise K; Wilkinson, Erica; Wilson, Marelle; Wilson, Monique; Moseley, G. Lorimer

---

VERSION 1 - REVIEW

**Reviewer**	**1**
**Name**	**Cacho-Díaz, Bernardo**
**Affiliation**	**Instituto Nacional de Cancerología, Neuro-oncology**
**Date**	**15-Sep-2025**
**COI**	**None**

This is a very manuscript describing a novel tool to evaluate the quality of systematic reviews whose aim is to analyze prognostic factors. Minor comments would be:

1- A figure to explain the use of the tool for inexperienced readers would be a ideal.

2. It is important to make differences between prognostic and predictive factors which are sometimes used indistinguishably during the manuscript; i.e., one depends on the treatment.

3. Individual methods for building prognostic models vary and there is no universal consensus although some guidelines (TRIPOD) have been published; this could be discussed a bit further.

Great work

**Reviewer**	**2**
**Name**	**Nelemans, Patty**
**Affiliation**	**Maastricht University, Epidemiology**
**Date**	**04-Nov-2025**
**COI**	**None**

This study addresses the level of agreement between assessments when using AMSTAR-PF, a standardized tool for rating the quality of systematic reviews of prognostic factors studies. Fourteen appraisers rated 8 articles. The authors used the Gwet's agreement coefficient (AC) for evaluation of evaluated interrater agreement (across 14 raters), inter-pair agreement (across 7 pairs of raters) and intrapair agreement (between two members of each of the 7 pairs). They also assessed usability by measuring the time needed for completion of the AMSTAR-PF appraisal process, performed several subgroup analyses and concluded that the AMSTAR-PF displays an acceptable level of usability and agreement.

The topic of this study is relevant. Many researchers attach high value to standardized tools for quality assessment, and it is important to know to what extent different raters arrive at the same judgement. After reading the manuscript, I have several questions and comments.

1. The conclusion relies heavily on the use and interpretation of Gwet's agreement coefficients (AC) instead of Cohen's and Fleiss kappa coefficients. It is a deviation from the original protocol (as admitted by the authors), because the authors followed statistical advice to use the Gwet's AC. I would appreciate a more detailed explanation why Gwet's AC better reflects the level of agreement between raters than the kappa coefficients. It is essential information, especially considering the paper by Vach et al. (reference 41 in the manuscript) which states that "Gwets's AC1 is not a substitute for Cohens's kappa and that the verbal classification of kappa values by Landis & Koch should not be applied".

2. A convincing explanation on the superiority of Gwet's AC is particularly important, because in this study, the mean Gwet's coefficients for individual AMSTAR-PF questions are mostly higher than the corresponding kappa coefficients and give a more favorable impression of the level of agreement. However, it is also noteworthy that there are almost no differences between Gwet's and kappa ACs for the final appraisal by raters. Interrater, inter-pair and intrapair Gwet's ACs are 0.46, 0.46 and 0.68 vs kappa ACs of 0.46, 047 and 0.65, respectively. For readers it would be interesting to understand why this is so. Can the authors explain this finding in the discussion?

3. It remains unclear which agreement measure (interrater agreement, inter-pair agreement or intrapair agreement) is considered as the primary outcome measure in this study. The decision to add interrater agreement (for extra detail) was made after the protocol was registered (page 6, lines 46-47). This suggests that originally the authors considered

interrater agreement as less important and only added it as an afterthought. This surprises me, because to me evaluation of interrater agreement seems deeply relevant for current scientific practice. Evaluation of intrapair agreement seems useful before undertaking quality appraisal of articles. The relevance for practice of evaluation of inter-pair agreement is not clear and requires more explanation.

4. Can the authors explain why throughout the manuscript, they pay much more attention to intrapair ACs than to interrater ACs? For instance, the description of results in the abstract and paragraph “Subgroup analysis” centers on comparison of mean intrapair Gwet’s AC between subgroups and there is little attention given to interrater agreement coefficients. However, the differences between interrater ACs for Cochrane systematic reviews and non-Cochrane reviews are even larger than those between the intrapair ACs. Mean Gwet’s interrater AC for the individual AMSTAR-PF questions are 0.82 vs 0.51, respectively. For final appraisal, the interrater ACs are 0.89 and 0.44. Why are these results not presented?

On page 8, lines 36-37, information is given that across the 56 intrapair agreements for overall quality 60.7% were identical. Why is this information not provided for interrater agreement, although it is truly relevant and would facilitate the interpretation of the interrater agreement coefficients?

5. The discussion does not address several relevant issues.

a) The discussion now begins with a general statement that agreement scores ranged from slight to almost perfect. It would be more informative to address the findings with respect to the point estimates for interrater and intrapair agreement coefficients. The mean Gwet’s interrater AC for the individual AMSTAR-PF questions (0.59) and for final appraisal (0.46) fall into a different category of the classification by Landis and Koch than the corresponding ACs for intrapair agreement (0.75 and 0.68). These findings should serve as the basis for the conclusion as to whether agreement scores can be considered acceptable.

b) My concern is that there is still considerable interrater variation which can lead to arbitrary over- or underestimation of the quality of systematic reviews. The conclusion at the end of the discussion that AMSTAR-PF displays an acceptable level of agreement seems to come out of the blue. The authors considered it necessary to make several modifications to individual AMSTAR-PF questions with low Gwet’s ACs (Table 1) and re-assessment of agreement scores of the modified AMSTAR-PF has not been performed.

c) The paragraph on comparison of the results with measures of agreement for other established tools (page 12, lines 9-23) does not help to reduce my concern. It mentions intrapair and inter-pair ACs of 0.00 and 0.07 for ROBINS-1 and 0.11 and 0.00 for ROB-NRSE. The point that the authors want to make is that AMSTAR-PF performs better than these other tools, but the presented results are not very reassuring and raise serious doubts about the usability of these other tools. To my opinion, these findings cannot be merely mentioned without further consideration.

d) The findings in the paragraph “Subgroup analysis” are not adequately discussed. The similarity of mean intrapair Gwet’s AC for individual questions for the first two articles versus the next six articles (0.76 vs 0.74) and for the first two articles versus the final two articles (0.76 vs 0.83) seems to indicate that there was no learning effect. Can the authors comment on this issue?

e) The significantly higher mean intrapair AC for the two Cochrane articles compared to the AC for the 6 non-Cochrane reviews (0.88 vs 0.71) as well as the large differences between the interrater ACs also require more detailed discussion. The findings are quite worrisome and raise doubts about the ability of raters to objectively assess the quality of reviews without being biased by the knowledge that the systematic review comes from the Cochrane Collaboration.

f) The use of Landis and Koch’s benchmarks for Gwet’s AC scores is considered as a potential limitation. The authors tried to mitigate the problem “by using standard errors to calculate cumulative probabilities, as recommended by Gwet” and here they refer to a handbook. However, this book is not easily accessible for most readers and more detailed explanation in the text of the manuscript would be nice. Furthermore, Fleiss and Cohen’s kappa coefficients are presented in the appendix and can also be used as benchmarks. Unfortunately, questions that arise from comparison of Gwet’s AC and kappa ACs are not discussed (see also comment 2).

Minor comment

The authors might reconsider the term “agreeability testing” in the title considering the meaning according to Internet: Agreeability testing measures a person's tendency to be cooperative, kind, and compassionate ….?

**Reviewer**	**3**
**Name**	**Dack, Kyle**
**Affiliation**	**University of Bristol, MRC Integrative Epidemiology Unit**
**Date**	**18-Nov-2025**
**COI**	**None**

Statistical review summary:

This is a very interesting manuscript, detailing the development of what would be a valuable new tool to evaluate prognostic factor reviews.

Generally, I have very little to suggest to improve the paper. The reporting is clear, the analysis uses valid methods and results are interpreted reasonably. I have just a few minor suggestions or queries.

Methods:

1. I suggest somewhere early in the methods reporting when the testing occurred.

2. Outcomes: It may help readers to add a section briefly describing the three agreement outcomes (interrater, inter-pair, intra-pair) and what utility they add - as some readers may not be familiar with all. This section could also elaborate on why interrater agreement was added post protocol.

Results:

3. The original AMSTAR is mentioned – it may be beneficial to briefly clarify the purpose of this tool and reference it (or perhaps in the introduction).

4. Table 1 and 2. I am unsure if pink is intuitive in the legend scale. The legend generally uses a diverging scale (red/brown on one end, green/blue on the other) and this is easy to understand. But pink is outside of this scale, so it is unclear from glancing whether pink is "better" or worse than red. It may be clearer if fair and moderate were represented by orange and yellow?

5. Table 1 and 2. The colour categories do not seem consistent with the benchmark legend. For example: Table 1, Item 1, interrater, has an AC of 0.70, 95% CI 0.49 to 0.91, but category of moderate (0.4 to 0.6).

Is the LCI used to determine category? Because in most cases it appears the LCI is consistent with the benchmark legend. If so, this should be reported in the methods and table descriptions.

However, I notice there are a few items where even the LCI does not align with the benchmark category. E.g. Table 1, item 14, interrater, and Table 2, item 2b, inter-pair.

If a different statistic was used to generate the categories, I recommend including this in each table to avoid reader confusion.

6. Subgroup analyses: Are the test statistics essential for reader interpretation e.g. t-statistic, Wilcoxan W? If not, I suggest removing them to improve readability and statistical clarity.

7. Table 3. It may be clearer if the order of columns 4 to 8 are swapped, i.e. begin with min, and with max.

VERSION 1 - AUTHOR RESPONSE

Reviewer: 1
Dr. Bernardo Cacho-Díaz, Instituto Nacional de Cancerología, Medica Sur
Comments to the Author:
This is a very manuscript describing a novel tool to evaluate the quality of systematic reviews whose aim is to analyze prognostic factors. Minor comments would be:
1- A figure to explain the use of the tool for inexperienced readers would be a ideal.

We have added a figure (Figure 1 in the revised manuscript; reproduced below) to provide more detail about the testing process and the different outcomes we assessed.

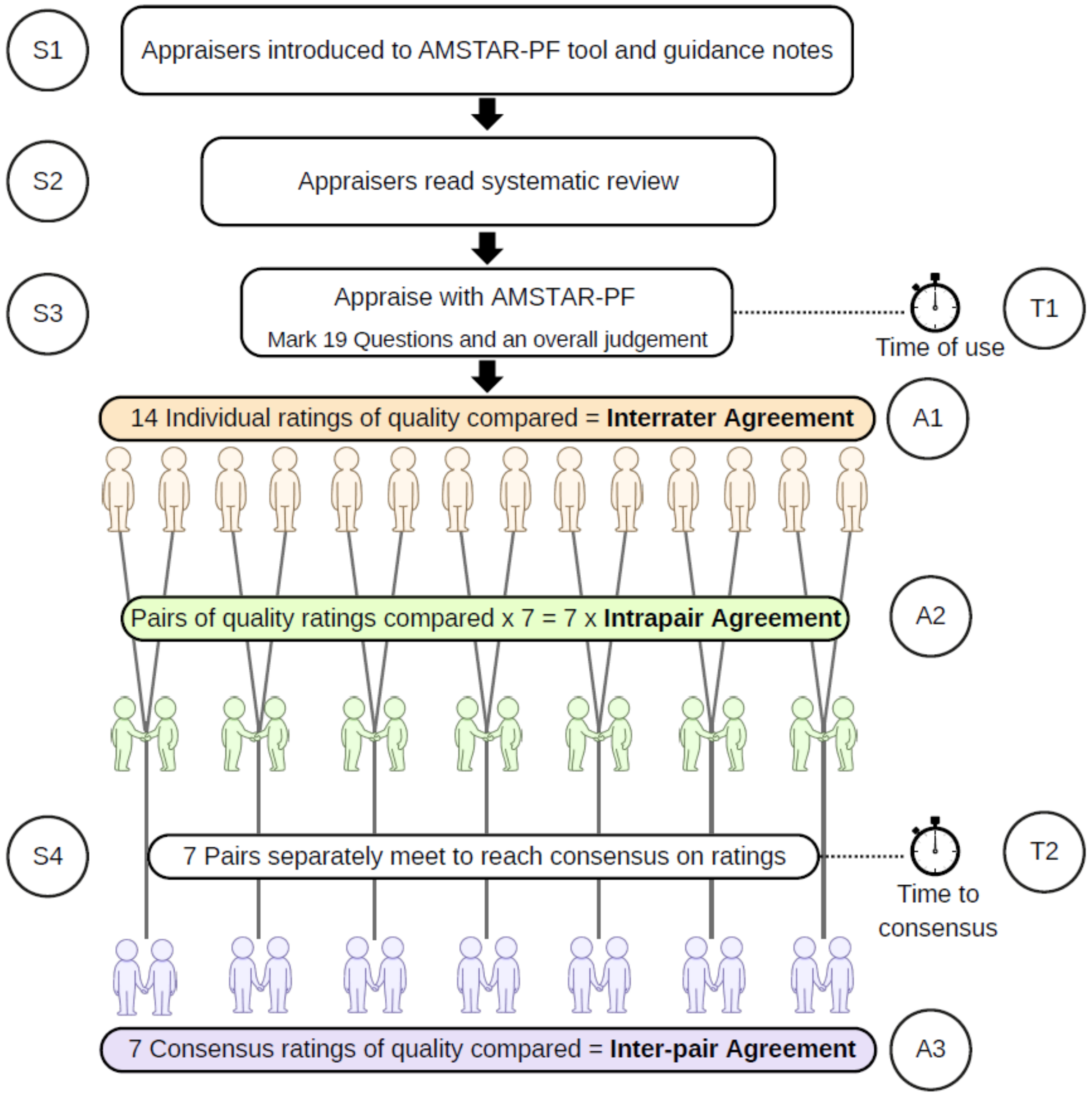

Figure 1: Schematic of testing procedure, showing the steps involved (S1-4) for appraisers, the types of agreement (A1-3), and the timings assessed (T1-2).

First, all appraisers were introduced to AMSTAR-PF **(S1)**. Each appraiser then read the systematic review **(S2)**. and timed themselves while they used AMSTAR-PF to appraise the article, completing all 19 questions and the overall judgment of review quality **(S3)**. This gave a **Time of use (T1)**. The ratings for each question and overall quality were compared across the 14 appraisers to give **Interrater Agreement (A1)**. These ratings were also compared within each of the seven pre-allocated pairs, to give seven scores of **Intrapair Agreement (A2)**. The pairs met up (after the first two articles, and then at the end), and timed themselves reaching consensus on each of the 19 questions and overall appraisal **(S4)**, giving a **Time to consensus (T2)**. The seven pairs' scores for the 19 questions and overall rating were compared to give **Inter-pair Agreement (A3)**.

2. It is important to make differences between prognostic and predictive factors which are sometimes used indistinguishably during the manuscript; i.e., one depends on the treatment.

Thanks for this comment; it is a good point, given that the preferred nomenclature varies in different fields. We have searched the manuscript and confirmed prognostic factors is used

consistently throughout. We have added the following italicised parts to the introduction (p 3, lines 13-20) to ensure that the scope and relevance of these results are clear:

PFs are variables associated with *(the risk or value) of future outcomes (3), and are particularly useful in providing patients with a prognosis and developing prognostic models (eg, for outcome risk prediction). Nomenclature for PFs is not consistent in the medical literature, and sometimes PFs are also known as predictors, risk factors or prognostic variables/determinants/covariates. Importantly, our focus is not on whether factors are predictive of treatment effect (sometimes called predictive markers, especially in the cancer field); rather, we are solely focused on factors that are associated with future outcomes (irrespective of how it impacts any treatment effect).*

3. Individual methods for building prognostic models vary and there is no universal consensus although some guidelines (TRIPOD) have been published; this could be discussed a bit further.

Thank you for this suggestion. We considered this suggestion at great length. Ultimately we concluded that discussion of methods of building prognostic models was beyond the scope of this paper. As you rightly mention, it is a complicated and difficult space, and we don't believe that we could do it justice in this paper, without adding a large number of words and taking the reader off topic. We think that future iterations of AMSTAR-PF could branch to include prognostic modelling, but given the differences between prognostic factor and modelling studies we believe a modified AMSTAR-PF dedicated to that kind of study, would be better.

Great work
Thanks kindly, we appreciate your review of our paper and well-considered suggestions.

Reviewer: 2
Miss Patty Nelemans, Maastricht University
Comments to the Author:
This study addresses the level of agreement between assessments when using AMSTAR-PF, a standardized tool for rating the quality of systematic reviews of prognostic factors studies. Fourteen appraisers rated 8 articles. The authors used the Gwet's agreement coefficient (AC) for evaluation of evaluated interrater agreement (across 14 raters), inter-pair agreement (across 7 pairs of raters) and intrapair agreement (between two members of each of the 7 pairs). They also assessed usability by measuring the time needed for completion of the AMSTAR-PF appraisal process, performed several subgroup analyses and concluded that the AMSTAR-PF displays an acceptable level of usability and agreement.

The topic of this study is relevant. Many researchers attach high value to standardized tools for quality assessment, and it is important to know to what extent different raters arrive at the same judgement. After reading the manuscript, I have several questions and comments.

Thank you for your comprehensive review and thoughtful feedback on our manuscript.

1. The conclusion relies heavily on the use and interpretation of Gwet's agreement coefficients (AC) instead of Cohen's and Fleiss kappa coefficients. It is a deviation from the original protocol (as admitted by the authors), because the authors followed statistical advice to use the Gwet's AC. I would appreciate a more detailed explanation why Gwet's AC better reflects the level of agreement between raters than the kappa coefficients. It is essential information, especially

considering the paper by Vach et al. (reference 41 in the manuscript) which states that "Gwets's AC1 is not a substitute for Cohens's kappa and that the verbal classification of kappa values by Landis & Koch should not be applied".

Thank you for this observation. To the first point, we would respectfully disagree that the conclusion is reliant on the use of Gwet's AC, rather than kappas. A conclusion based on kappa scores would be very similar, albeit based on statistics we consider to be less appropriate, as detailed further below. The overall appraisal agreement using kappa benchmarks are either moderate (interrater, inter-pair) or substantial (intrapair); all are higher than the Gwet's AC benchmarks. Similar to the results using Gwet's AC, the results using kappa are similar to or better than other published examples when comparing kappas (see p 13, lines 4-8) in the discussion: *Published benchmarked agreements when using Kappa range from no agreement to moderate agreement for a range of tools, including AMSTAR 2 (33, 37, 38), ROBIS (33, 37-39), ROB 2 (Revised Cochrane Risk of Bias tool for randomised trials (40))(41, 42), ROBINS-I (43), and PROBAST (Prediction model Risk Of Bias ASsessment Tool (44, 45))*. So the conclusion that AMSTAR-PF could be considered appropriate and useful, given broader standards in the field and levels of agreement and time of use of extant tools, remains equally valid regardless of which agreement measure is used.

Measuring agreement is a debated and controversial topic to which we gave much consideration, in collaboration with statisticians and diving deep into the extant literature. We have sought to add more detail to the paper, whilst being aware that a full discussion of the theory, assumptions and mathematics underscoring various agreement statistics remains outside the scope and limits of a paper such as ours, so have added more references for readers to explore. We have also included more detailed explanation in these responses.

The main reason that many authors prefer Gwet's AC is its superior stability and interpretability when the prevalence of a category is very high or very low. This is relevant for a scenario such as ours, where it is important to have accurate measures of agreement in high or low agreement situations – knowledge that is important for a tool's use.

We agree with Vach et al. insofar as we do not think Gwet's AC and Cohen's Kappa are direct substitutes. While both are used as agreement, it has been argued that Gwet's AC is an **agreement** test, whereas Cohen's Kappa is an **association** test (Silveira & Siqueira 2023). Notwithstanding that they are often used similarly, because our aim was to test agreement of scores, rather than association, Gwet's AC seemed most appropriate.

We had originally sought to avoid verbal classification of Gwet's AC, following our reading of Vach et al. To our knowledge, however, there is no specific verbal classification system that has been developed, other than that proposed by Gwet using the cumulative probabilities alongside Landis & Koch's labels. Having no verbal interpretation of Gwet's AC seemed more problematic, as it could leave readers who do not have detailed knowledge of Gwet's AC with little understanding of what the numbers represent, and potentially *overestimating* the agreement if they were to just transfer the point estimates directly into a classification, such as Landis & Koch's, without any context or adjustment for uncertainty. The concept of verbal rating systems in general has also been criticised (eg, Vanbelle et al., 2025), but remains a common measure to present alongside statistics. Again, despite significant investigation, we are still not aware of any alternatives to a verbal rating system to aid interpretation of results.

The following example illustrates why Gwet's AC provides a more appropriate measure of agreement than kappa. If we consider the following tables (representing the answers to Q7a). The left-hand table shows the 14 appraisers as columns, and the 8 papers as rows, with answers coded as 1=Y, 2=PY, 3=PN, 4=N. The right-hand table shows the consensus scores of each pair as columns. Intuitively both tables look to have a high degree of agreement. In the left-hand table, the percentage agreement is 87.5%, and Gwet's AC is 0.8219. Fleiss' kappa is 0.1234. For the consensus scores in the right-hand table, the percentage agreement is 87.7%, Gwet's AC is 0.8247, and Fleiss' kappa 0.1001. Benchmarks for both tables are 'substantial' agreement for Gwet's AC, and 'slight' agreement for kappa.

		Individual Appraisers															Pairs						
		1	2	3	4	5	6	7	8	9	10	11	12	13	14		1	2	3	4	5	6	7
Article Number	1	1	1	1	1	1	1	1	1	1	1	1	1	1	1		1	1	1	1	1	1	1
	2	2	2	1	1	1	1	1	1	1	1	4	1	1	1		2	1	1	1	1	1	1
	3	2	2	1	1	1	1	1	1	1	1	1	1	1	1		2	1	1	1	1	1	1
	4	2	2	1	1	1	2	1	2	2	2	2	2	1	1		2	1	2	1	2	2	1
	5	2	2	2	1	1	1	2	3	1	1	2	2	1	1		2	2	1	3	1	2	1
	6	1	1	1	1	1	1	1	1	1	1	1	1	1	1		1	1	1	1	1	1	1
	7	1	1	1	1	1	1	2	2	1	1	2	2	1	1		1	1	1	2	1	2	1
	8	2	1	1	1	1	1	1	2	1	1	2	2	1	1		1	1	1	2	1	2	1

Similarly, in the collapsed data (Y/PY = 1; N/PN = 4) for question 7a (below), the left-hand table shows the 14 individual raters' scores as columns, and the right-hand table shows the seven consensus scores as the seven columns (the 8 rows are the different articles in both tables). Intuitively, this looks like very good agreement, and the percentage agreement is 96.4% for both tables. Gwet's AC is 0.9630 for interrater and inter-pair agreement ('almost perfect' agreement). Fleiss' kappa, however, is -0.0182 for both interrater and inter-pair, ie, agreement that is *worse than chance* for both ('poor' agreement). This is an example of the kappa paradox – there is a high level of agreement but a very low kappa score for agreement – and why Gwet's AC is seen by many as a more appropriate representation of agreement. Intuitively, it is difficult to look at the tables below and conclude that agreement is worse than chance, or 'poor'. 'Almost perfect' agreement appears a much more appropriate interpretation of the results.

		Individual Appraisers															Pairs						
		1	2	3	4	5	6	7	8	9	10	11	12	13	14		1	2	3	4	5	6	7
Article Number	1	1	1	1	1	1	1	1	1	1	1	1	1	1	1		1	1	1	1	1	1	1
	2	1	1	1	1	1	1	1	1	1	1	4	1	1	1		1	1	1	1	1	1	1
	3	1	1	1	1	1	1	1	1	1	1	1	1	1	1		1	1	1	1	1	1	1
	4	1	1	1	1	1	1	1	1	1	1	1	1	1	1		1	1	1	1	1	1	1
	5	1	1	1	1	1	1	1	4	1	1	1	1	1	1		1	1	1	4	1	1	1
	6	1	1	1	1	1	1	1	1	1	1	1	1	1	1		1	1	1	1	1	1	1
	7	1	1	1	1	1	1	1	1	1	1	1	1	1	1		1	1	1	1	1	1	1
	8	1	1	1	1	1	1	1	1	1	1	1	1	1	1		1	1	1	1	1	1	1

Ultimately, kappa has a longer history, has been more commonly used, was our initial planned measure, and will likely be familiar to many readers. However, we consider Gwet's AC to be a better measure of agreement, more stable across a range of values, and it is becoming more

commonly used. By presenting both, but favouring what we (and many others) consider to be the more appropriate measure, we allow readers to appraise all the information and focus on the measures with which they are most comfortable.

Again, we thank you for raising the point and the opportunity to respond. We have included the following additions in the manuscript:

In the methods:

P5, lines 35-36: [Gwet's AC]… *and is becoming more recommended in a range of settings (16-25).* We have added another 7 references (Zec et al., 2017, Konstantinidis et al., 2022, Cibulka & Strube, 2021, Dettori & Norvell, 2020, Klein 2018, Popplewell et al., 2018, Wongpakaran et al., 2013) here which detail the more appropriate results using Gwet's AC over Cohen's/Fleiss kappa, and/or recommend the use of Gwet's AC rather than kappa in different fields and settings.

P5, lines 38-39: *Furthermore, although Kappa and Gwet's AC are used in agreement testing, it has been argued that Cohen's Kappa is an association test, rather than an agreement test (26).*

In the discussion:

P13, lines 17-35: *Although both the Gwet's AC and kappa values were comparable to other tools, it is worth noting differences between the two. Gwet's AC values were generally higher than the kappa values. This is a common finding (20, 25, 46) because kappa is reported to overestimate chance agreement (16, 47). The well-recognised kappa paradox (17, 18) occurred in questions where agreement was high, with low kappa values leading to large differences noted between Gwet's AC and kappa. Question 7a illustrates this paradox and the more appropriate values of Gwet's AC. With all answering options, the percentage of agreement across the 14 appraisers is 87.5%, Gwet's AC is 0.82 ('substantial' agreement), yet Fleiss' kappa is 0.12 ('slight' agreement). The percentage agreement is 87.7% across the 7 pairs' consensus scores, with Gwet's AC 0.82 ('substantial') and Fleiss' kappa 0.10 ('slight'). The results when comparing the collapsed answering options are even more extreme; 96.4% agreement (110/112 individual scores agreed, 55/56 consensus scores agreed), Gwet's AC 0.96 ('almost perfect' agreement), and Fleiss' kappa -0.02 ('poor' agreement – with the negative value suggesting agreement worse than chance). In situations where agreement was lower, for example the agreement scores of the overall rating, Gwet's AC and kappa are more closely aligned (interrater 0.46 vs 0.46, inter-pair 0.46 vs 0.47, and intrapair 0.68 vs 0.65, for Gwet's AC vs kappa respectively). The differences in behaviour of the agreement scores with different prevalences, and familiarity of different readers with different agreement measures, may point to a benefit in presenting multiple measures to allow for a nuanced picture of agreement levels and facilitate comparability across studies (48).*

Additional references:

Silveira PSP, Siqueira JO. Better to be in agreement than in bad company. Behavior Research Methods. 2023;55(7):3326-47.

Vanbelle S, Engelhart CH, Blix E. Measuring Agreement in Diagnostics: A Practical Guide for Researchers. Statistics in Medicine. 2025;44(23-24):e70299

Zec S, Soriani N, Comoretto R, Baldi I. High Agreement and High Prevalence: The Paradox of Cohen's Kappa. The Open Nursing Journal. 2017;11.

Konstantinidis M, Le L, Gao X. An Empirical Comparative Assessment of Inter-Rater Agreement of Binary Outcomes and Multiple Raters. Symmetry. 2022;14:262.

Cibulka M, Strube M. The Conundrum of Kappa and why some Musculoskeletal Tests Appear Unreliable despite High Agreement: A Comparison of Cohen Kappa and Gwet's AC to Assess Observer Agreement when Using Nominal and Ordinal Data. Physical therapy. 2021.

Dettori J, Norvell D. Kappa and Beyond: Is There Agreement? Global Spine Journal. 2020;10:499-501.

Klein D. Implementing a General Framework for Assessing Interrater Agreement in Stata. The Stata Journal. 2018;18:871-901.

Popplewell M, Reizes J, Zaslawski C. Appropriate Statistics for Determining Chance-Removed Interpractitioner Agreement. The Journal of Alternative and Complementary Medicine. 2018;25(11):1115-20.

Wongpakaran N, Wongpakaran T, Wedding D, Gwet KL. A comparison of Cohen's Kappa and Gwet's AC1 when calculating inter-rater reliability coefficients: a study conducted with personality disorder samples. BMC Medical Research Methodology. 2013;13(1):61.

2. A convincing explanation on the superiority of Gwet's AC is particularly important, because in this study, the mean Gwet's coefficients for individual AMSTAR-PF questions are mostly higher than the corresponding kappa coefficients and give a more favorable impression of the level of agreement. However, it is also noteworthy that there are almost no differences between Gwet's and kappa ACs for the final appraisal by raters. Interrater, inter-pair and intrapair Gwet's ACs are 0.46, 0.46 and 0.68 vs kappa ACs of 0.46, 047 and 0.65, respectively. For readers it would be interesting to understand why this is so. Can the authors explain this finding in the discussion?

Thank you for this observation. In general, the higher the percentage of agreement, the more likely that the kappa paradox will be seen – abnormally low kappa ratings of agreement (and illustrated above in our response to your first point). In more mid-range values, the results of kappa and Gwet's AC will be more similar, as is observed in the overall appraisal agreements.

Please see the response above for what we have included in the manuscript (specifically p13, lines 17-35).

3. It remains unclear which agreement measure (interrater agreement, inter-pair agreement or intrapair agreement) is considered as the primary outcome measure in this study. The decision to add interrater agreement (for extra detail) was made after the protocol was registered (page 6, lines 46-47). This suggests that originally the authors considered interrater agreement as less important and only added it as an afterthought. This surprises me, because to me evaluation of interrater agreement seems deeply relevant for current scientific practice. Evaluation of intrapair agreement seems useful before undertaking quality appraisal of articles. The relevance for practice of evaluation of inter-pair agreement is not clear and requires more explanation.

We did not nominate a primary outcome measure during the planning of the study; we felt that in different situations different measures will be of interest to different researchers and readers

and merely sought to present findings. As you've correctly noted, the 14-way interrater agreement was not in the original protocol. We took an approach to the measures that was reflective of our understanding of what the tool was likely to be commonly used for – evaluating systematic reviews as part of an umbrella review – and what is considered good practice in such a situation. Because best practice recommends quality appraisal to be completed in duplicate, we are of the opinion that any individual's rating is less important than the consensus rating arrived at by a pair of reviewers – the consensus rating is usually presented in a publication, the ratings of the individual reviewers are not. However, knowing how different pairs of reviewers (for example, different umbrella reviews using overlapping systematic reviews) would rate the same article does seem of clear value, so the inter-pair agreement was a priority. Similarly, from a practical perspective, how much disagreement each pair had seems relevant for a team undertaking quality appraisal. We could not, practically, think of a situation where the agreement scores of 14 individual reviewers would likely to be important, so had not prioritised that. Prior to data analysis, however, we saw that it would be an easy additional step that provides some extra information, so decided to calculate and present this to add further detail. As observed by the reviewer, we clearly note this as a deviation from protocol.

We have added the following (p 5, lines 18-27):

We chose the analyses based on measures that we believed would be most important for potential users of the tool. A common reason for appraising the quality of a systematic review is as part of an umbrella review. In most umbrella reviews, quality appraisal will be performed independently by two reviewers, with the consensus score being the final, accepted rating of quality. As such, the intrapair agreement is likely to be relevant for review teams in most situations, and the inter-pair agreement is most relevant for comparing different review teams who have assessed the same paper. The interrater agreement was added as another metric but is perhaps less practically relevant – it is rarer that a single person's appraisal will be published, and so comparing many different individuals' ratings would then be less likely.

4. Can the authors explain why throughout the manuscript, they pay much more attention to intrapair ACs than to interrater ACs? For instance, the description of results in the abstract and paragraph "Subgroup analysis" centers on comparison of mean intrapair Gwet's AC between subgroups and there is little attention given to interrater agreement coefficients. However, the differences between interrater ACs for Cochrane systematic reviews and non-Cochrane reviews are even larger than those between the intrapair ACs. Mean Gwet's interrater AC for the individual AMSTAR-PF questions are 0.82 vs 0.51, respectively. For final appraisal, the interrater ACs are 0.89 and 0.44. Why are these results not presented?
On page 8, lines 36-37, information is given that across the 56 intrapair agreements for overall quality 60.7% were identical. Why is this information not provided for interrater agreement, although it is truly relevant and would facilitate the interpretation of the interrater agreement coefficients?

As mentioned above (in question 3), we saw relatively little benefit in detailed analysis of interrater agreement across 14 individuals, simply because it is a situation very unlikely to ever occur in practice. Systematic reviews perhaps differ in this regard to other situations, such as assessing clinical tests, where a single clinician's or laboratory's results will be used and compared, as opposed to consensus scores. Authors undertaking a review are likely to want to understand intrapair agreement, because in most cases two appraisals will be performed and

that concordance (or divergence) is pertinent, and sometimes presented in publications. Similarly, knowing how the consensus scores of different pairs agree is of great importance, as the consensus score is generally the final verdict on the review's quality and what would be presented in a paper. In this respect, our inter-pair agreement is perhaps more equivalent to what interrater agreement represents in many clinical or laboratory situations.

With regards to presenting percentages for the intrapair agreements, we provided this information because it was easily interpretable. Review pairs have an easily interpretable binary outcome – identical or different (or, identical +/- one category vs not). For the interrater agreement of the overall rating, for instance, there are only 8 decisions (1 for each paper). Because there are 14 ratings to compare, it is much less readily translatable. With 14 raters, there was not 100% agreement across all 14 raters for any of the eight papers. So, although we could report 0/8 were identical, we don't believe it adds much information. If there was an even split between all four quality categories, this would be reported the same as if 13/14 scored it the same, or if half nominated the highest and half nominated the lowest category. We could provide a percentage of agreement, however that is generally not recommended because it doesn't differentiate when agreement may have happened by chance – which is the underlying basis for the development (and some of the controversy) behind other agreement scores, such as the various kappas, and agreement coefficients. We have aimed throughout to provide a range of different information, and are happy to explore different ways of presenting this information if you have further suggestions.

In terms of the subgroup analyses, we weren't able to give interrater agreement for some, due to the counterbalanced order of completion. That is, six individuals did one order, and eight did the reverse, so it is impossible to compare the agreement on the first article across 14 raters because they are different articles. A similar difficulty occurs with inter-pair ratings. It would be possible to analyse each stream separately, then take an average (perhaps proportionate to the number of appraisers in each stream, because they're not equal), but we do not believe that this facilitates interpretation. By using intrapair, we can give this information, as each member of each pair completed the same order of articles. Furthermore, because after two appraisals were completed each pair met separately (as opposed to all 14 appraisers meeting) we believe there is greater interest in whether a difference in intrapair agreement was observed pre- and post the second article – that is, did the consensus meeting change how similarly paired appraisers scored articles.

We considered presenting the Cochrane vs non-Cochrane article agreement for interrater and inter-pair in the manuscript, but because it's not a primary measure and the results were consistent with the presented intrapair data, we did not believe it a priority, figuring the appendix would suffice (especially because we couldn't assess interrater and inter-pair for all the subgroups – we wanted to harmonise our approach to the measures presented in the manuscript section).

5. The discussion does not address several relevant issues.

a) The discussion now begins with a general statement that agreement scores ranged from slight to almost perfect. It would be more informative to address the findings with respect to the point estimates for interrater and intrapair agreement coefficients. The mean Gwet's interrater AC for the individual AMSTAR-PF questions (0.59) and for final appraisal (0.46) fall into a different category of the classification by Landis and Koch than the corresponding ACs for

intrapair agreement (0.75 and 0.68). These findings should serve as the basis for the conclusion as to whether agreement scores can be considered acceptable.

Thank you for this suggestion. We have added (p12, lines 30-34):

Interrater agreement averaged 0.59 (range, 0.21 to 0.90), inter-pair agreement 0.61 (range 0.24 to 0.91) and intrapair agreement 0.75 (range 0.45 to 0.95) across the domains, with agreement for the overall rating 0.46 (95%CI 0.30 to 0.62) for interrater agreement, 0.46 (95%CI 0.17 to 0.74) for inter-pair agreement, and 0.68 (range of averages 0.22 to 1.00) for intrapair agreement.

In the absence of any guidelines relating to benchmarks for acceptable agreement, we relied on published data about other tools; tools that are considered acceptable by the field via their endorsement (eg, Cochrane or other guidelines) and repeated use. We discuss the comparison with other tools a little later (see responses below), while also highlighting variability in agreement scores and performance.

b) My concern is that there is still considerable interrater variation which can lead to arbitrary over- or underestimation of the quality of systematic reviews. The conclusion at the end of the discussion that AMSTAR-PF displays an acceptable level of agreement seems to come out of the blue. The authors considered it necessary to make several modifications to individual AMSTAR-PF questions with low Gwet's ACs (Table 1) and re-assessment of agreement scores of the modified AMSTAR-PF has not been performed.

There is considerable interrater variability in AMSTAR-PF, and in all such tools we reviewed. It is a challenge for the field, and one that is unlikely to have an easy solution. Our conclusion that AMSTAR-PF is acceptable is not solely based on the interrater agreement, however. There are, to our knowledge, no published benchmarks for what constitutes an acceptable tool. Rather, (as mentioned above), we were informed by published agreement and time of use data from other tools that have been endorsed and widely used. We agree that there is likely over- and underestimation of the quality of reviews, but also that some variation in the literature is likely due to what different teams value as 'quality', which may depend on their training, backgrounds, biases, and the purpose of their quality appraisal and broader aims. Furthermore, as with our response to point 5e) below, we would differentiate between the accuracy of the quality judgement and the level of agreement. Perfect agreement scores do not necessarily mean that the review has been accurately assessed, just that the appraisers judged it the same. We have not directly tested the accuracy of the quality judgements in this paper, although it stands to reason that more consistent appraisal scores are more likely to be near the 'true' quality of the paper, and wildly varied ones may increase the risk of less accurate judgements.

We were not able to complete another full round of testing after making final modifications – the time and personnel burden was not feasible. The changes made were relatively minor, and although we believe they are important and will assist future users, did not represent a large change to the nature of the tool. The appendix clearly details the changes made to the questions, so readers can review that information and factor those changes into their understanding of the tool and testing.

c) The paragraph on comparison of the results with measures of agreement for other established tools (page 12, lines 9-23) does not help to reduce my concern. It mentions

intrapair and inter-pair ACs of 0.00 and 0.07 for ROBINS-1 and 0.11 and 0.00 for ROB-NRSE. The point that the authors want to make is that AMSTAR-PF performs better than these other tools, but the presented results are not very reassuring and raise serious doubts about the usability of these other tools. To my opinion, these findings cannot be merely mentioned without further consideration.

We have added the following in the discussion to discuss this (p13, lines 8-16):

As illustrated here, there is significant variability in published agreement scores for a range of tools, and the agreement is generally modest. The variance in scores may be a result of different teams of appraisers, papers, and interpretations, amongst other variables. The low agreement scores highlight the difficulties and subjectivity inherent to appraising articles. There is unlikely to be an easy or obvious replacement to quality appraisal tools. Increased use of reporting guidelines is one area that is likely to improve the agreement seen on quality appraisal tools – having a clear indication of what was performed (and how) decreases the subjectivity of appraisers in making their judgements, and although this won't always improve the quality of the review, it can allow for more harmonious judgements by appraisers.

d) The findings in the paragraph "Subgroup analysis" are not adequately discussed. The similarity of mean intrapair Gwet's AC for individual questions for the first two articles versus the next six articles (0.76 vs 0.74) and for the first two articles versus the final two articles (0.76 vs 0.83) seems to indicate that there was no learning effect. Can the authors comment on this issue?

Thank you for highlighting this gap. We have added the italicised portion of the paragraph below (p14, lines 8-20):

"Familiarity with the tool (and perhaps prognostic factor research and systematic reviews more generally) may reduce time to complete appraisals and gain consensus. Usability data showed improved timing as people progressed through the allocated papers, irrespective of the order of completion. *Interestingly, there was no significant change in agreement scores over the course of the testing. This suggests that learning effects may occur in efficiency, but have little impact on how studies are scored. The exploratory comparison of the Cochrane vs non-Cochrane articles found that Cochrane articles showed higher levels of agreement. This is unsurprising, as Cochrane articles are reported to have higher standards of reporting, which may make answering questions easier as the information is more explicit. Appraisers were not, however, blinded to the publication details, so may have also assumed the information they were seeking was recorded and more consistently and thoroughly looked for it. Future testing of appraisal tools may consider removing details such as journal name, author details and institutions, to allow for blinded appraisal, and pre-specifying this as an outcome of interest."*

e) The significantly higher mean intrapair AC for the two Cochrane articles compared to the AC for the 6 non-Cochrane reviews (0.88 vs 0.71) as well as the large differences between the interrater ACs also require more detailed discussion. The findings are quite worrisome and raise doubts about the ability of raters to objectively assess the quality of reviews without being biased by the knowledge that the systematic review comes from the Cochrane Collaboration.

It is an interesting and valuable point. We did not pre-plan this analysis, and the testers were therefore blind that this would be a subgroup to be tested. In hindsight, it would be interesting to extract solely the text of the articles, and remove publication and author details – we thank you for this suggestion; it is something we'll consider for future research. We would like to differentiate, however, between the quality scores given to the Cochrane articles, and the agreement scores between reviewers. It is possible that reviewers were biased to err more favourably knowing that it was a Cochrane review, and hence score more generously than if they thought it wasn't a Cochrane publication, but it is potentially less likely that their biases would have led them to construct a higher level of agreement. For instance, if two reviewers felt a certain question was ambiguous, it is possible that they might each be tempted to mark the question as probably yes, knowing it was a Cochrane review. If so, the opposite is also likely true; knowing an article wasn't Cochrane could lead to the temptation to mark the ambiguous question as probably no, which would harmonise agreement for the non-Cochrane articles to an equivalent degree. For this reason we are of the opinion that the agreement scores are likely less influenced than the quality scores. In fact, given there is a range of literature that reports Cochrane articles are generally more thorough in design, conduct, and (importantly) reporting, it would be perhaps more worrisome if no difference was found in quality as this may imply the tool didn't discriminate between high and lower quality studies. Similarly, an article with clearer reporting is likely to remove ambiguity and facilitate easier, more confident quality appraisal, which would be reflected in more similar marking. Looking back at the data, this was borne out – 86.5% of the Cochrane domains were answered with the more definite Y, N, N/A options, and 13.5% with the "probably" options; whereas 61.8% of non-Cochrane article appraisals had the more definite options, and 38.2% had probably options. Again, it is possible that this was a reflection of positive bias towards Cochrane articles (and negative bias towards non-Cochrane articles), but it seems less likely that appraisers consistently misinterpreted ambiguous reporting as definite in the Cochrane articles, and definite statements as ambiguous in the non-Cochrane articles. Whether blinding would change agreement scores remains unanswered – ultimately, blinding appraisers to publication details would help answer this question, and would be recommended for future work. It should be cautioned, however, that this would make the agreement data less directly applicable to real world situations, because quality appraisal for umbrella (or systematic) reviews is unlikely to be performed blinded to publication detail.

As described in the response to point 5d), above, we have added the following (p14, lines 13-20): *The exploratory comparison of the Cochrane vs non-Cochrane articles found that Cochrane articles showed higher levels of agreement. This is unsurprising, as Cochrane articles are reported to have higher standards of reporting, which may make answering questions easier as the information is more explicit. Appraisers were not, however, blinded to the publication details, so may have also assumed the information they were seeking was recorded and more consistently and thoroughly looked for it. Future testing of appraisal tools may consider removing details such as journal name, author details and institutions, to allow for blinded appraisal, and pre-specifying this as an outcome of interest*.

f) The use of Landis and Koch's benchmarks for Gwet's AC scores is considered as a potential limitation. The authors tried to mitigate the problem "by using standard errors to calculate cumulative probabilities, as recommended by Gwet" and here they refer to a handbook. However, this book is not easily accessible for most readers and more detailed explanation in the text of the manuscript would be nice. Furthermore, Fleiss and Cohen's kappa coefficients are presented in the appendix and can also be used as benchmarks. Unfortunately, questions

that arise from comparison of Gwet's AC and kappa ACs are not discussed (see also comment 2).

We have added an additional reference (Klein 2018 – see section 2.8, pp879-881) that describes the rationale and method of using standard errors to calculate cumulative probabilities. While not the original source, it is readily accessible, so will provide interested readers who can't access Gwet's handbook with an alternative overview of this information.

We have added the following italicised parts to the methods (p6, lines 2-8): "This manner considers the standard error to give a probability of the value falling within a certain benchmark category, *rather than simple alignment of the point estimate*. We used the recommended 95% cumulative probability as the cut-off level (27). *To calculate this, the probability of the agreement falling within each benchmark band is calculated, and the probabilities summed starting with the top band (0.8-1.0) and working down. Once the cumulative probability reaches 95% or higher then that band is the one used as the benchmark interval (23, 27).*"

As discussed above, in comment 2, we have added a paragraph in the discussion to compare AC and kappa (p13, lines 17-35).

Minor comment
The authors might reconsider the term "agreeability testing" in the title considering the meaning according to Internet: Agreeability testing measures a person's tendency to be cooperative, kind, and compassionate ....?

We have made this modification for the title and throughout.

Reviewer: 3
Dr. Kyle Dack, University of Bristol
Comments to the Author:
Statistical review summary:
This is a very interesting manuscript, detailing the development of what would be a valuable new tool to evaluate prognostic factor reviews.
Generally, I have very little to suggest to improve the paper. The reporting is clear, the analysis uses valid methods and results are interpreted reasonably. I have just a few minor suggestions or queries.

Thank you for your review and valuable feedback

Methods:
1. I suggest somewhere early in the methods reporting when the testing occurred.

Thanks for this comment; we added, "*in March 2024*", to the methods section (p4, line 23).

2. Outcomes: It may help readers to add a section briefly describing the three agreement outcomes (interrater, inter-pair, intra-pair) and what utility they add - as some readers may not be familiar with all. This section could also elaborate on why interrater agreement was added post protocol.

Thank you. As detailed for the third comment of reviewer 2, above, we took an approach to the measures reflective of our understanding of what the tool was likely to be commonly used for –

evaluating systematic reviews as part of an umbrella review – and what is considered good practice in such a situation. Because best practice recommends quality appraisal to be completed in duplicate, we are of the opinion that any individual's rating is less important than the consensus rating arrived at by a pair of reviewers – the consensus rating is usually presented in a publication, the ratings of the individual reviewers are not. However, knowing how different pairs of reviewers (for example, different umbrella reviews using overlapping systematic reviews) would rate the same article does seem of clear value, so the inter-pair agreement was a priority. Similarly, from a practical perspective, how much disagreement each pair had seems relevant for a team undertaking quality appraisal. We could not, practically, think of a situation where the agreement scores of 14 individual reviewers would likely to be important, so had not prioritised that. Prior to data analysis, however, we saw that it would be an easy additional step that provides some extra information, so decided to calculate and present this to add further detail.

We have made an additional figure (Figure 1, also reproduced above in response to Reviewer 1, point 1) to help explain the process and show visually the different outcomes, and have added the following in the text (p 5, lines 18-27):

We chose the analyses based on measures that we believed would be most important for potential users of the tool. A common reason for appraising the quality of a systematic review is as part of an umbrella review. In most umbrella reviews, quality appraisal will be performed independently by two reviewers, with the consensus score being the final, accepted rating of quality. As such, the intrapair agreement is likely to be relevant for review teams in most situations, and the inter-pair agreement is most relevant for comparing different review teams who have assessed the same paper. The interrater agreement was added as another metric but is perhaps less practically relevant – it is rarer that a single person's appraisal will be published, and so comparing many different individuals' ratings would then be less likely.

Results:
3. The original AMSTAR is mentioned – it may be beneficial to briefly clarify the purpose of this tool and reference it (or perhaps in the introduction).

We have now referenced AMSTAR 2 in the introduction (p3, line 31-32): *AMSTAR-PF was based on AMSTAR 2 (6), a quality appraisal tool designed for systematic reviews of interventional research*.

4. Table 1 and 2. I am unsure if pink is intuitive in the legend scale. The legend generally uses a diverging scale (red/brown on one end, green/blue on the other) and this is easy to understand. But pink is outside of this scale, so it is unclear from glancing whether pink is "better" or worse than red. It may be clearer if fair and moderate were represented by orange and yellow?

This is a good point, and one we considered during the construction of the graphs and tables. The main limiting factor for us was the graphs; we wanted to use the background colours to illustrate the bands of the benchmarks, and yet be able to clearly superimpose the values of the point estimates and confidence intervals over the top, indicating the likely benchmark category using 95% cumulative probability (see the response to point 5, below, for more detail). Five pairs of clearly different shades were therefore required. We have constructed an alternative version, which we believe is more intuitive but still allows for differences in benchmarks to be visualised. An example of a graph and a table are reproduced below:

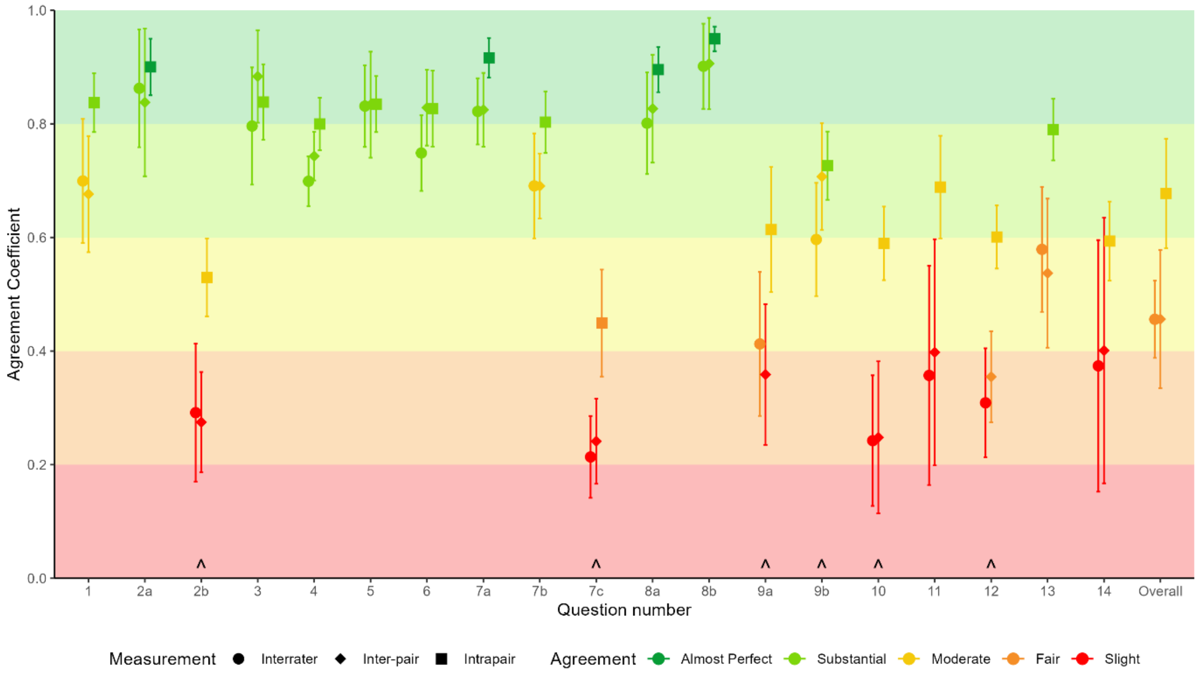

Question	Interrater AC	(95% CI)	I	Inter-pair AC	(95% CI)	I	Intrapair AAC	(95% CI)	I
1 Research Question	0.70	(0.44 - 0.96)		0.68	(0.43 - 0.92)		0.84	(0.71 - 0.96)	
2a Protocol Registration	0.86	(0.62 - 1.00)		0.84	(0.53 - 1.00)		0.90	(0.78 - 1.00)	
^2b Deviations from protocol	0.29	(0.00 - 0.58)		0.27	(0.07 - 0.48)		0.53	(0.36 - 0.70)	
3 Included study designs	0.80	(0.55 - 1.00)		0.88	(0.69 - 1.00)		0.84	(0.68 - 1.00)	
4 Search strategy	0.70	(0.60 - 0.80)		0.74	(0.64 - 0.84)		0.80	(0.67 - 0.91)	
5 Inclusion process	0.83	(0.66 - 1.00)		0.83	(0.61 - 1.00)		0.83	(0.71 - 0.96)	
6 Excluded studies	0.75	(0.59 - 0.91)		0.83	(0.67 - 0.99)		0.83	(0.66 - 0.99)	
7a Data extraction	0.82	(0.68 - 0.96)		0.82	(0.67 - 0.98)		0.92	(0.83 - 1.00)	
7b Description of studies	0.69	(0.47 - 0.91)		0.69	(0.55 - 0.83)		0.80	(0.67 - 0.94)	
^7c PF effect calculations	0.21	(0.04 - 0.38)		0.24	(0.06 - 0.42)		0.45	(0.22 - 0.68)	
8a RoB process	0.80	(0.59 - 1.00)		0.83	(0.60 - 1.00)		0.90	(0.80 - 0.99)	
8b RoB technique	0.90	(0.72 - 1.00)		0.91	(0.72 - 1.00)		0.95	(0.90 - 1.00)	
^9a Synthesis interpretability	0.41	(0.11 - 0.71)		0.36	(0.07 - 0.65)		0.61	(0.34 - 0.88)	
^9b Meta-Analysis	0.60	(0.36 - 0.83)		0.71	(0.48 - 0.93)		0.73	(0.58 - 0.87)	
^10 Small study effects	0.24	(-0.03 - 0.51)		0.25	(-0.07 - 0.56)		0.59	(0.43 - 0.75)	
11 Impact of RoB	0.36	(-0.10 - 0.81)		0.40	(-0.07 - 0.87)		0.69	(0.47 - 0.91)	
^12 Heterogeneity	0.31	(0.08 - 0.54)		0.35	(0.17 - 0.54)		0.60	(0.46 - 0.74)	
13 Conflicts of interest	0.58	(0.32 - 0.84)		0.54	(0.23 - 0.85)		0.79	(0.66 - 0.92)	
14 Certainty of findings	0.37	(-0.15 - 0.90)		0.40	(-0.15 - 0.95)		0.59	(0.42 - 0.76)	
**Final Appraisal**	**0.46**	**(0.30 - 0.62)**		**0.46**	**(0.17 - 0.74)**		**0.68**	**(0.44 - 0.91)**	

Table 1: **Gwet's AC for each AMSTAR-PF question, with all answering options, and for the final judgement**. Questions marked (^) had an N/A option and were treated as nominal data, calculated as Gwet's AC1. All other questions were calculated as Gwet's AC2 with linear weighting. Confidence intervals are capped at 1.00. Benchmark interpretation is calculated using 95% cumulative probabilities for Landis and Koch's benchmark categories, < 0, Poor; 0.0-0.2, Slight; 0.2-0.4, Fair; 0.4-0.6, Moderate; 0.6-0.8, Substantial; and 0.8-1.0, Almost Perfect.

AC, Gwet's Agreement Coefficient; Avg, average; I, Interpretation category; 95% CI, 95% Confidence Interval.

5. Table 1 and 2. The colour categories do not seem consistent with the benchmark legend. For example: Table 1, Item 1, interrater, has an AC of 0.70, 95% CI 0.49 to 0.91, but category of

moderate (0.4 to 0.6).
Is the LCI used to determine category? Because in most cases it appears the LCI is consistent with the benchmark legend. If so, this should be reported in the methods and table descriptions.
However, I notice there are a few items where even the LCI does not align with the benchmark category. E.g. Table 1, item 14, interrater, and Table 2, item 2b, inter-pair.
If a different statistic was used to generate the categories, I recommend including this in each table to avoid reader confusion.

Thank you for highlighting this. The colours correspond to the 95% cumulative probability of an agreement placing in a particular category, not where the point estimate or the lower CI is. The location of the point estimate will sometimes correspond to the category when there is a relatively constrained CI, and sometimes align with the lower 95%CI, but not always. See Gwet's (2014) handbook and Klein 2018 (section 2.8, pp879-881) for more information.

We have added the following italicised parts to the methods (p6, lines 2-8): "This manner considers the standard error to give a probability of the value falling within a certain benchmark category, *rather than simple alignment of the point estimate*. We used the recommended 95% cumulative probability as the cut-off level (27). *To calculate this, the probability of the agreement falling within each benchmark band is calculated, and the probabilities summed starting with the top band (0.8-1.0) and working down. Once the cumulative probability reaches 95% or higher then that band is the one used as the benchmark interval (23, 27).*"

6. Subgroup analyses: Are the test statistics essential for reader interpretation e.g. t-statistic, Wilcoxan W? If not, I suggest removing them to improve readability and statistical clarity.

We have made this change.

7. Table 3. It may be clearer if the order of columns 4 to 8 are swapped, i.e. begin with min, and with max.

Thank you for this suggestion, we have made the suggested change.
